# Computer-Aided Bleeding Detection Algorithms for Capsule Endoscopy: A Systematic Review

**DOI:** 10.3390/s23167170

**Published:** 2023-08-14

**Authors:** Ahmmad Musha, Rehnuma Hasnat, Abdullah Al Mamun, Em Poh Ping, Tonmoy Ghosh

**Affiliations:** 1 Department of Electrical and Electronic Engineering, Pabna University of Science and Technology, Pabna 6600, Bangladesh; musha.160217@s.pust.ac.bd (A.M.); rehnumahasnat.160234@s.pust.ac.bd (R.H.); 2 Faculty of Engineering and Technology, Multimedia University, Melaka 75450, Malaysia; mamun130203@gmail.com; 3 Department of Electrical and Computer Engineering, The University of Alabama, Tuscaloosa, AL 35487, USA; tghosh@crimson.ua.edu

**Keywords:** bleeding classification, bleeding detection, bleeding recognition, bleeding segmentation, capsule endoscopy, wireless capsule endoscopy

## Abstract

Capsule endoscopy (CE) is a widely used medical imaging tool for the diagnosis of gastrointestinal tract abnormalities like bleeding. However, CE captures a huge number of image frames, constituting a time-consuming and tedious task for medical experts to manually inspect. To address this issue, researchers have focused on computer-aided bleeding detection systems to automatically identify bleeding in real time. This paper presents a systematic review of the available state-of-the-art computer-aided bleeding detection algorithms for capsule endoscopy. The review was carried out by searching five different repositories (Scopus, PubMed, IEEE Xplore, ACM Digital Library, and ScienceDirect) for all original publications on computer-aided bleeding detection published between 2001 and 2023. The Preferred Reporting Items for Systematic Review and Meta-Analyses (PRISMA) methodology was used to perform the review, and 147 full texts of scientific papers were reviewed. The contributions of this paper are: (I) a taxonomy for computer-aided bleeding detection algorithms for capsule endoscopy is identified; (II) the available state-of-the-art computer-aided bleeding detection algorithms, including various color spaces (RGB, HSV, etc.), feature extraction techniques, and classifiers, are discussed; and (III) the most effective algorithms for practical use are identified. Finally, the paper is concluded by providing future direction for computer-aided bleeding detection research.

## 1. Introduction

The small bowel (SB) in humans has a complex looped-shape configuration and an extremely large length (around 6 m). For SB disease diagnosis, endoscopy can be used to detect tumors, cancer, bleeding, and Crohn’s disease [1]. In 2001, capsule endoscopy (CE, also called wireless capsule endoscopy, WCE) was approved by the Food and Drug Administration in the United States. It is a noninvasive technology that was primarily designed to provide diagnostic imaging of the SB, as this part of the human body is difficult to inspect through instrumental examination. CE represents the latest endoscopic technique that has revolutionized the treatment and diagnosis of diseases of the upper gastrointestinal (GI) tract, SB, and colon. A CE device consists of a complementary metal-oxide-semiconductor (CMOS) camera sensor with a microchip, a light-emitting diode (LED), a radiofrequency (RF) transmitter, and a battery. A clinical examination involving the use of CE can be executed in an ambulatory or hospital setting on an outpatient basis. After fasting overnight (8–12 h), a small capsule is swallowed by the patient. The capsule provides a wireless circuit and micro-imaging video technology is used for the acquisition and transmission of images. Software that localizes the device during its passage through the intestine is encompassed in the system. The capsule is propelled by peristaltic movement when it goes through the SB. While moving along the GI tract, images are captured at a fixed frame rate (2 frames per second, fps), although the newest model of CE manufactured by Given Imaging (PillCam SB 3 capsule) is able to achieve a frame rate of 2–6 fps based on the capsule speed as it travels through the SB [2]. These images are transferred to a data recorder worn on a belt outside the patient’s body, and about eight hours after swallowing, the patient returns to the clinic where the data and images are downloaded. Within 24–48 h, the capsule is passed through the patient’s stool.

CE is considered a first-line examination tool for diagnosing various kinds of diseases, including ulcers, polyps, bleeding, and Crohn’s disease [3]. A single scan may include up to 10,000 images of the GI tract for each patient, but evidence of abnormalities may appear in only a few of them. A very common abnormality found in the GI tract is bleeding [4]. To detect this, many researchers have contributed high-performance classifiers. Detection of bleeding at an early age is critical since it is a precursor for inflammatory bowel diseases such as Crohn’s disease and ulcerative colitis (UC). Bleeding is not only limited to the stomach; it can occur anywhere in the GI tract [5]. It is considered to be a common abnormality detected by CE and is often defined as “bleeding of unknown origin that recurs or persists or is visible after an upper endoscopy and/or negative endoscopy result” [6]. The major challenge is that residual traces and blood spots do not have typical shapes or textures, and their colors can range from light red to dark intense red and brown, making it difficult to distinguish blood from other digestive contents. This diversity of color might depend on the position of the camera, the bleeding timing [7], and the neighboring conditions of the intestinal content [8]. Bleeding is not a single pathology, and it may be caused by a variety of small intestinal diseases, such as open wounds, vascular lesions, angiodysplasia, ulcers, Crohn’s disease, and tumors. To discriminate the pathology, both texture and color features are used.

Since the CE diagnostic process captures over 57,000 images, manual reviewing is a labor-intensive and time-consuming task for physicians in order to detect bleeding regions [9], and it may involve several challenges due to complex backgrounds, low contrast, variations in the lesion, and color. This may affect the accuracy of subsequent classification and segmentation [10,11]. These issues complicate objective disease diagnosis and necessitate the opinions of many specialists to avoid misdiagnosis.

As a result, there is a strong need for an alternate technique to automatically detect bleeding in the GI tract. Some research has been conducted on the automated inspection and analysis of CE images. Software suites that use computational techniques are often made available with the brand of a particular capsule and are used by a lot of people. The benefits include the efficiency and availability of a tool that can automatically detect bleeding regions and improve diagnostic accuracy. Commercial software built by Given Imaging aims to recognize spontaneous active blood, although the reported sensitivity and specificity are not satisfactory [8]. Although CE has many advantages, research in CE technology is not widespread. For instance, at present, it is challenging for physicians to go through the entire collection of more than 50,000 frames in order to diagnose a disease. Due to visual fatigue and the relatively small size of the lesion region, the disease may go undetected in its early stages. The fact that software packages already available on the market are based on low-level, hand-crafted feature extraction algorithms that have poor generalizability should not be ignored. Additionally, because the feature extraction and classification phases are separated in hand-crafted feature-based techniques, it is difficult to make reliable diagnostic decisions.

Several informative original articles and reviews on bleeding detection in CE images have been published over the last 15 years. The authors of [12] reviewed the clinical applications and developments of small bowel CE, i.e., small bowel tumors, Celiac disease, and Crohn’s disease. They gave insight into the potential future prospects of small bowel CE. In [13], the authors discussed different imaging methods, including signal processing, color and image processing, and artificial intelligence, for representing, analyzing, and evaluating CE images. In [14], the authors calculated performance metrics (accuracy, positive and negative predictive values, sensitivity, specificity) and compared the diagnostic accuracy of video CE and double-balloon enteroscopy in cases of obscure GI bleeding of vascular origin. Another study [15] discussed the market’s available CE models, including diagnostic yield, safety profile, image quality, and technical evolution, for small bowel CE. That study analyzed five commercially available types of small bowel capsule endoscopes, which were the PillCam^®^SB2, MiroCam^®^v2, EndoCapsule^®^, OMOM^®^ (SmartCapsule), and CapsoCam^®^SV1. In [16], the authors reviewed and analyzed the literature for computational methods that could be applied in software to improve the diagnostic yield of video CE. Another research group [17] reviewed a deep learning-based approach (CNN) for CE, which was used to solve a variety of issues, e.g., detection of polyp/ulcer/cancer, bleeding/hemorrhage/angiectasia, and hookworms. In [18], the authors reviewed all of the image features (color, texture, shape) for image abstraction in machine vision-based analysis of CE videos and reviewed computer-aided diagnostic systems of CE images. The authors mainly concentrated on the study of shot boundaries and GI pathology detection. In the literature, the authors of [19] assessed the accuracy of video CE to identify active hemorrhage in the upper GI. In another article [20], the authors discussed deep learning methods (CNN-based algorithms) for WCE, in which only the PubMed repository was used for article selection. Moreover, none of these review articles particularly focused on only bleeding detection algorithms for CE. The foremost contributions of this paper are summarized as follows:

1. A taxonomy for computer-aided bleeding detection algorithms for capsule endoscopy is identified.
2. Various color space and feature extraction techniques are used to boost the bleeding detection performance, which is discussed in depth.
3. From the observation of the existing literature, direction for the computer-aided bleeding detection research community is provided.

The emphasis of this work is only on state-of-the-art bleeding detection algorithms using CE, which differentiates this paper from various recent review papers. This review was performed by gathering the required information from recent research and organizing it according to taxonomy, analyzing the performance of bleeding detection methods, and providing a path for future research. Moreover, in order to improve the current acceptance of computer-aided bleeding detection algorithms in CE, it is hoped that this effort will capture advanced techniques that will be more acceptable in real-life applications.

## 2. Review Methodology

The Preferred Reporting Items for Systematic Review and Meta-Analyses (PRISMA) [12] guidelines were followed for this review. The authors separately screened the titles and abstracts of the publications retrieved through the database search and then carried out a full-text review of all relevant studies. This methodology used the following processes.

### 2.1. Identifying Research Question

To conduct this systematic review, one research question was selected:

**Available state-of-the-art computer-aided bleeding detection algorithms for capsule endoscopy: How accurate and suitable are they for practical use?**

The answer to this question will help to improve computer-aided bleeding detection algorithms for CE and identify the research gaps in the current methodology.

### 2.2. Database

Comprehensive searches for suitable literature were performed across five repositories: Scopus, PubMed, IEEE Xplore, ACM Digital Library, and ScienceDirect.

### 2.3. Search Strategy

To cover all of the approaches for bleeding detection in CE, the considered keywords were: ‘Bleeding’, ‘Hemorrhage’, ’Blood’, ‘Detection’, ‘Segmentation’, ‘Recognition’, ‘Classification’, and ‘Capsule Endoscopy’. The search query string was: (“Bleeding” OR “Hemorrhage” OR “Blood”) AND (“Detection” OR “Segmentation” OR “Recognition” OR “Classification”) AND (“Capsule Endoscopy”).

The search results were confined to the English language. The primary references of the selected full-text articles were analyzed for related publications. Articles related to non-humans, posters, and book chapters were excluded. Articles that fulfilled the exclusion criteria, shown in Table 1, were filtered out.

### 2.4. Results

**Figure 1:**
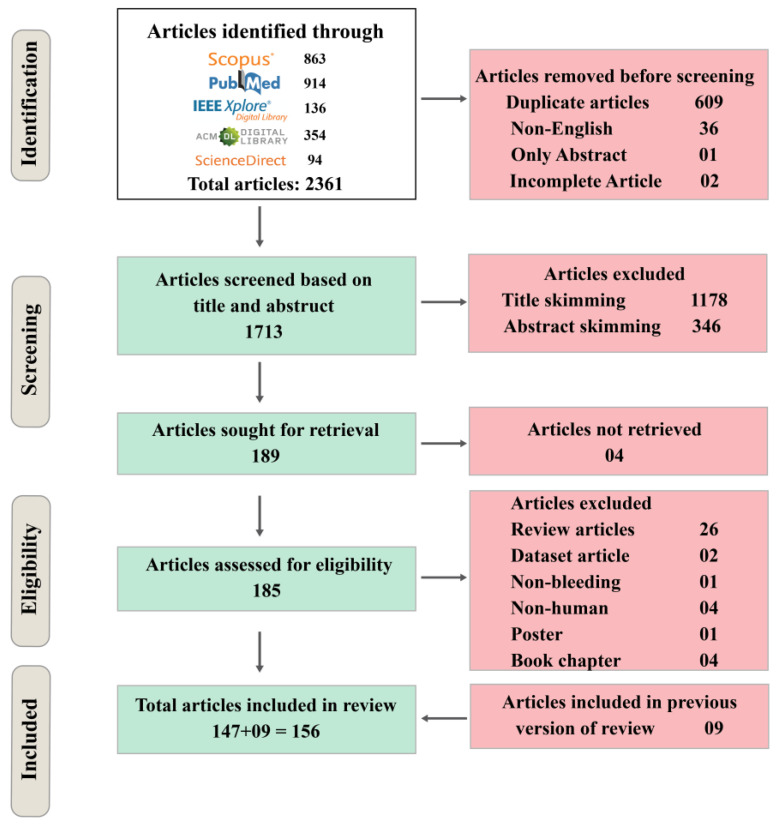
Flowchart of the inclusion and exclusion processes of this systematic review.

The initial search resulted in a total of 2361 publications. A total of 609 duplicates, 36 non-English articles, one abstract, and two incomplete articles were removed before the screening. Based on the abstracts and titles, 1713 articles were set aside for screening. A total of 1178 articles were excluded through title skimming and 346 were excluded through abstract skimming. Four articles were not retrieved. As a result, 185 articles were included as eligible articles, from which 26 review articles, 2 dataset articles, 1 non-bleeding articles, 4 non-human articles, 1 poster, and 4 book chapters were excluded. Through the process, 156 articles were included in the systematic review after including 9 published review articles. Figure 1 shows the methodology and results of the systematic review.

## 3. Review Findings

### 3.1. Taxonomy

**Figure 2:**
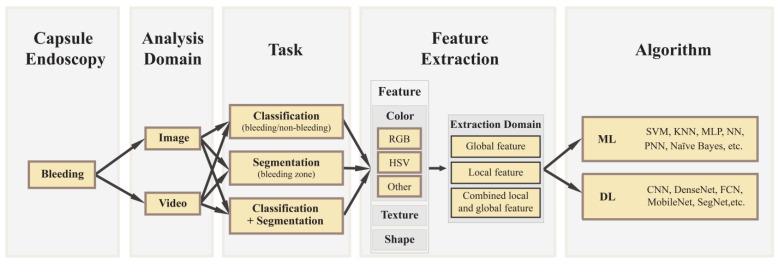
Taxonomy of Computer-Aided Bleeding Detection Algorithms for Capsule Endoscopy.

Based on the current literature, the CE datasets comprised two types according to the domain analysis: image and video. The literature based on CE image and video datasets was included in this review. To answer the research question, a taxonomy was generated from the literature findings, which is shown in Figure 2. The image and video domains were further divided into three categories: classification, segmentation, and combined (classification + segmentation). Classification is a task that can be performed to classify bleeding images from non-bleeding images using classification algorithms. To detect a bleeding zone, segmentation is another important technique that can cluster a bleeding image into several coherent subregions. Both classification and segmentation algorithms are used in the development of advanced computer-aided diagnosis systems to identify bleeding images as well as recognize bleeding regions. Different types of feature extraction techniques, for example, color space, shape, texture attributes with region of interest (ROI), pixel- or block-wise contourlet transform, etc., featured extraction domains that were used to tune the machine learning algorithms for accurate identification of the bleeding images. Also, various convolutional neural networks, such as Visual Geometry Group (VGG), Residual Network (ResNet), Densely-Connected Convolutional Networks (DenseNet), etc., were used to extract features from CE images. Most of the literature used a color space for feature extraction, like RGB (Red, Green, and Blue), HSV (Hue Saturation Value), HIS (Hue, Saturation, Intensity), YCbCr (Luminance, Chrominance [chroma CB and chroma CR]), CMYK (Cyan, Magenta, Yellow, and Key [black]), CIE L*a*b* (Lightness [L], Red—Green [a], and Yellow—Blue [b]), etc. From the review findings, the state-of-the-art computer-aided bleeding detection algorithms were categorized into two types: conventional machine learning algorithms, such as Support Vector Machine (SVM), K-Nearest Neighbors (KNN), K-Means Clustering, Naïve Bayes, Random Tree, Random Forest, Artificial Neural Networks (ANN), Probabilistic Neural Networks (PNN), Multilayer Perceptron (MLP), etc., and deep learning algorithms, such as CNN, AlexNet, VGG, ResNet, SegNet, DenseNet, etc. The available bleeding detection algorithms in the literature covered the research question.

### 3.2. Analysis Domain

Two types of CE datasets of bleeding were available. One was image domain datasets, and the other was video domain datasets. Most of the literature reviews proposed bleeding detection systems based on the image domain.

#### 3.2.1. Image

An image is a matrix of pixels organized in columns and rows. The grayscale image represents a one-dimensional matrix and the RGB image represents a three-dimensional matrix. Based on the characteristics of the image, a digital machine or processor can analyze the medical image to detect the abnormality. Over eight hours, a CE captured approximately 55,000–57,000 images throughout the GI tract in one experiment. In [21], the authors proposed a CE image dataset obtained from 10 patients at the University of Malay Medical Center (UMMC). The dataset consisted of 100 bleeding and 300 normal images, which had a resolution of 288 × 288 pixels. A total of 1131 bleeding lesions and 2164 normal non-sequential images were utilized by an algorithm to detect bleeding. The size of the images was 320 × 320 or 512 × 512 pixels [22]. In [23], the authors suggested the KID Dataset 2, which contained a total of 2352 CE images (bleeding: 303) with a resolution of 360 × 360 pixels, and MICCAI 2017, which contained a total of 3895 CE images (bleeding: 1570) with 320 × 320 or 512 × 512 resolution. A total of 1200 CE images with a resolution of 576 × 576 pixels were utilized in the study [24]. According to [25], the model was trained using 2000 images that were extracted from 20 different videos. The image dataset was available on capsuleendoscopy.com. An OMOM capsule produced 3596 CE images with 256 × 240 pixels size, which were extracted from five subjects [26]. A dataset of CE images with a size of 240 × 240 × 3 pixels, which contained 148 bleeding and 152 inactive images from 60 CE video snippets of 12 subjects, was utilized in a study [27].

#### 3.2.2. Video

Few studies utilized the CE video domain for automatic bleeding detection. The frames of the video are sequentially arranged and applied to an algorithm for identifying bleeding frames or regions. Time is an important parameter of the video domain. The video domain has the best likelihood of detecting bleeding by using the previous and next frames of the video as well as the time information. However, lengthy videos require high processing power. The authors of [28] utilized a video CE dataset with a sequence of 600 frames, which was collected from a PillCam SB3 video. Among them, 73% of the total frames were red lesions. In [29], if the last frame of the CE video was not found, the authors presented a pixel-based approach using the Support Vector Classifier method in which the model continued the process to the next frame of the video. In [30], a novel, full reference, video quality metrics method named Quality Index for Bleeding Regions in Capsule Endoscopy (QI-BRiCE) was proposed, which evaluated the perceptual and diagnostic qualities of damaged WCE videos with bleeding regions. Videos of 15 patients ranging in duration from 12,000 to 20,000 frames were used for automatic bleeding detection [31]. A semi-automatic method was proposed to extract the bleeding region from successive video frames containing bleeding. Three video files were used that included 589, 500, and 428 frames [32]. Deeba et al. improved the model by skipping one or a few frames from the sequence of bleeding frames [33]. A study utilized a CNN-based model to screen high-risk suspicious images from CE videos with a focus on high sensitivity but potentially lower specificity [34]. Using 84 full-length videos, another study [35] proposed an algorithm that was comparable with the Suspected Blood Indicator (SBI).

Five publicly accessible CE bleeding videos were tested in [36,37] to construct automatic bleeding detection models to identify bleeding. Ten real patient video files were used in [38], which consisted of 200 frames and about 40 s in duration. Instead of dealing with a complete video, consecutive small portions of videos were used. Each video consisted of several frames, which were tested sequentially [39]. Another study used the time domain information of CE videos for a bleeding localization technique. The approach used eight different videos collected from eight subjects at West China Hospital [40]. A total of 4166 third-generation small bowel CE videos were applied in [41], which were collected from the Computer-Assisted Diagnosis for Capsule Endoscopy database (CAD-CAP) endorsed by the Société Française d’Endoscopie Digestive.

#### 3.2.3. Task

In this review, all kinds of literature on the computer-aided bleeding detection approach were divided into three categories based on the task. The first category was the classification task for bleeding images or frames, the second was the segmentation task for bleeding zone identification, and the third was the combination of the classification and segmentation tasks.

##### Classification

Classification is a supervised learning technique in both machine learning and deep learning that is used to categorize a given set of data into classes. A classification model learns from the given dataset and then classifies new observations into a number of classes or groups, such as 0 or 1, yes or no, bleeding image or non-bleeding image, etc. The study in [23] presented a machine learning algorithm to classify CE images into two categories, named bleeding and non-bleeding images. Rustam et al. developed a deep neural network for the classification of bleeding CE images [42]. A video frame classification model using SVM, which is a machine learning algorithm, was presented in [30] to classify bleeding frames in CE videos.

##### Segmentation

Segmentation is a technique in which an image is broken down into different subregions according to the extracted features. An image is a collection or set of different pixels. Similar types of pixels are grouped according to image segmentation. This technique helps in minimizing the complexity of the image in order to simplify further processing or analysis of the image. E. Tuba et al. [43] presented an automated segmentation technique based on SVM to detect bleeding regions in CE images. In other research on bleeding zone segmentation of CE images, a deep learning-based model, named Multi-Stage Attention-Unet, was proposed [44]. A time domain-based segmentation approach was provided by W. Shi et al. [40] for locating bleeding in CE videos.

##### Classification + Segmentation

This category includes the classification and segmentation techniques at the same time to classify images and locate the region of similar types of pixels. In this review, several articles proposed to classify bleeding images and also to detect the bleeding region at the same time using both classification and segmentation techniques for the development of advanced computer-aided diagnosis systems. Rathnamala et al. presented a model based on Gaussian mixture model superpixels and SVM for automatic bleeding detection using CE images. First, the model classified bleeding and non-bleeding images, and then it applied the post-segmentation technique to detect the bleeding region in the bleeding image [45]. Two deep learning CNN-based models, AlexNet and SegNet, were presented in [46] to classify bleeding images and zones in CE images. In [36], a computer-aided scheme was presented for the classification of bleeding frames from CE videos, and then the post-segmentation technique was applied for the localization of the bleeding zone.

## 4. Feature Extraction

Features are a major part of any pattern recognition task. Feature extraction is the process of converting raw data (like an image) into a set of features. It aids in reducing the number of resources required to explain big amounts of data. Color and texture are two common and crucial image recognition properties. Both are also highly beneficial in extracting features from CE images to identify bleeding because bleeding areas have more or fewer color differences and/or textures compared with their neighboring environment. The feature extraction domain is a selection process that selects a region of the image that is used to efficiently extract features. Various feature extraction domains were used to accurately extract bleeding features, such as ROI, specific block, pixel level, and image level.

**Color Space:** Color space refers to a specific color arrangement. There are many different color spaces, such as RGB, HSV, YIQ, YCbCr, CMYK, CIE L*a*b*, CIE XYZ, etc. From the literature review, the color spaces used for feature extraction were categorized into four groups: RGB, HSV, Other, and Combined color spaces.

**RGB:** Images are represented in the RGB color space as an m-by-n-by-3 numeric array, the components of which indicate the intensity levels of red, green, and blue color channels. The range of numeric values is determined by the image’s data type. Different types of the RGB color space are available, such as linear RGB, sRGB (standard red, green, blue), adobe RGB, and so on. In CE images or videos, a bleeding zone is distinguished by the presence of a bright red or dark red zone. Many studies utilized the RGB color space to extract features for the identification of bleeding images or regions from CE images. The studies in [47,48] presented an automated obscure bleeding detection technique on the GI tract based on statistical RGB color features that could classify bleeding and non-bleeding images in CE images. By using the same RGB components of each pixel of the CE image, the study in [49] presented a system to automatically detect bleeding zones in CE images. From the first-order histogram in the RGB plane, the approach extracted bleeding color information from CE image zones by calculating the mean, standard deviation, skew, and energy [50]. Zhao et al. presented a two-dimensional color coordinate system in the RGB color space to segment abnormality in CE videos. The approach combined two descriptors to extract features: the first was based on image color content, while the second was based on image edge information [38]. Another research group proposed using color vector similarity coefficients to evaluate the color similarity in the RGB color space in order to detect bleeding in CE images [51]. Yun et al. presented a method using color spectrum transformation (CST) for the identification of bleeding in CE images. This approach included a parameter compensation step that used a color balance index (CBI) in the RGB color space to compensate for irregular image conditions [52]. The study in [53] suggested an automatic bleeding image detection technique utilizing an RGB color histogram as a feature extractor and bit-plane slicing to detect bleeding and non-bleeding images from CE videos. In [54], the authors utilized superpixel segmentation in RGB color format to extract bleeding information for an automatic obscure bleeding detection technique. In [21], an automated bleeding detection approach was presented using a color-based per-pixel feature extraction technique. Ghosh et al. [37] presented an automatic bleeding detection approach based on an RGB color histogram of block statistics to extract features from CE videos. To reduce computational complexity and flexibility, the approach utilized blocks of surrounding pixels rather than individual pixel values. The conventional machine learning model (which includes different stages: image acquisition, pre-processing, feature extraction, and classification) used single pixels for the training and testing data. Therefore, the model was unable to eliminate a few very small judged bleeding zones that were not bleeding. To address this issue, cluster of pixels-based feature extraction techniques have been used in some research to extract features from bleeding CE images. A cluster of pixels in the RGB color space was utilized instead of single pixels in an automatic bleeding classification system, which improved the sensitivity [55].

Instead of directly using the RGB color space, a G/R composite color plane was utilized to extract features from CE images in [36]. In other research, T. Ghosh et al. [39] extracted statistical features from the overlapping spatial blocks in CE images based on the G/R color plane. The R/G transform color plane pixel intensity ratio was utilized for the extraction of bleeding information from CE images [56]. Rather than considering individual pixels, Ghosh et al. considered the surrounding neighborhood block of the individual pixel and the R/G plane ratio for bleeding feature extraction from CE images [57]. Shi et al. [40] used a temporal red-to-green ratio (R/G) feature value to detect bleeding regions.

According to T. Ghosh et al. [58], the average pixel intensity ratio in the RGB color space was used to extract features from CE images for an automatic bleeding detection approach. The study in [59] presented rapid bleeding detection in CE videos. The red ratio (RR) in the RGB color space was used to extract a feature from each superpixel of CE images. Also, the RR feature for individual pixels was utilized for feature extraction of bleeding from CE images [25]. The various coefficients of the RGB color space for bleeding and non-bleeding superpixel blocks are RG (Red, Green), RB (Red, Blue), and GB (Green, Blue) in two-dimensional space. Liu et al. [60] presented an automatic gastric hemorrhage detection system based on the coefficient of variation in the RG two-dimensional color space for different superpixel blocks in CE images. Another transformation form of the RGB color space is the OHTA color space. In [61], the OHTA color space was utilized to extract the features of bleeding from CE images.

A custom RGB color space was proposed in [31], which was similar to the CMYK color space and was used to extract features for automatic blood detection in CE videos. Kundu et al. [62] presented a normalized RGB color space histogram-based feature extraction method to identify bleeding in CE images. Another research group employed a two-stage saliency map extraction method to localize the bleeding areas in CE images. The first-stage saliency map was constructed using a color channel mixer, and the second-stage saliency map was derived from the RGB color space’s visual contrast [63].

Some studies applied algorithms in the RGB color space to extract features from CE images or videos. Using advanced pattern recognition techniques, a MapReduce framework was presented for the identification of bleeding frames and segmentation of bleeding zones. For classification, the system encoded RGB color space information from the raw data of CE images using a K-means clustering algorithm, and for segmentation of the bleeding zone, a density-based algorithm (DBSCAN) was utilized [64]. Hwang et al. presented an automatic bleeding region detection system using the Expectation Maximization (EM) clustering algorithm in the RGB color space [65].

**HSV**: HSV (hue-H, saturation-S, value-V) is an alternative representation of the RGB color space that correlates better with the human perception system. The HSV color space is generated from cartesian RGB primaries, and its components and colorimetry are related to the color space from which it is derived. Several studies [1,11,29,35,43,46,66,67,68,69,70,71,72,73,74,75,76,77,78,79,80,81,82,83,84,85,86,87] employed the HSV color space histogram as the color feature descriptor to extract features from CE images or videos in automated bleeding detection systems. In [78], the RGB image was transformed into HSV color and several statistical parameters, like variance, kurtosis, skewness, entropy, etc., were calculated from the histograms of CE images. The extracted features were applied in a bleeding detection method. The contrast, cluster shade, cluster prominence, and entropy were computed to extract bleeding features from the Gray Level Co-occurrence Matrix (GLCM) in the HSV color space [79]. Usman et al. suggested a pixel-based method for the detection of bleeding regions in CE videos. The HSV color space was utilized to compute the bleeding information [29]. The study in [88] presented a color-based segmentation using the HSV color space to detect bleeding regions in CE images. Giritharan et al. presented a bleeding detection method based on the HSV color space with dominant color and co-occurrence of dominant colors for feature extraction to classify bleeding lesions [66]. In [67], the HSV color moments were used to extract bleeding features from CE images, achieving the highest accuracy compared to the local binary pattern (LBP), local color moments, and Gabor filter. Using a block-based color saturation approach in the HSV color space, CE images were classified as bleeding or non-bleeding in [68]. A two-stage analysis system was proposed in [69], in which both block- and pixel-based color saturation methods used the HSV color space to extract bleeding features. Color saturation and hue were obtained in the study by converting the input videos or images to the HSV color space. In [70], the authors compared texture features with color features extracted from the HSV color space for the classification of bleeding and showed that color features provided better results. A fuzzy logic edge detection technique was applied in the HSV color space in [1] to extract features of bleeding and non-bleeding from CE images. According to [9], a feature selection strategy was proposed based on HSV color transformation to extract geometric features from CE images for classifying bleeding images. To extract the color features, another research group [70] applied the HSV color space Scale Invariant Feature Transform (HSV-SIFT) for CE abnormality detection.

Various statistical features are computed from the hue, saturation, and value channels of the HSV color space. The hue space (H) provides a useful feature for color objects or surfaces. The hue space was utilized in [71] to extract features from CE images for an automatic bleeding detection approach. Another strategy used the combination of a hue–saturation (HS) color histogram with relevant features (64 bins) for extraction of information in order to identify suspected blood abnormalities [72]. The HSI (hue-H, saturation-S, intensity-I) color space is another variation of the HSV color space. A binary feature vector in the HSI color space was more effective in extracting features in a bleeding detection approach [73]. A segmentation approach in which the average saturation from the HSI color space, as well as the skewness and kurtosis of the uniform LBP histogram, were used as features for automated segmentation to detect bleeding in CE images [43]. Another research group suggested an HSI color histogram to follow a moving background and bleeding color distributions through time in the first stage. Cui et al. [74] presented six color features in the HSI color space to classify bleeding and normal CE images.

**Other color spaces:** In addition to the most popular RGB and HSV color spaces, a few articles utilized different color spaces to extract bleeding features from CE images, such as YIQ (luminance-Y, chrominance-IQ: in phase-I and quadrature-Q), YCbCr, CIE L*a*b*, CIE XYZ, and K-L (Karhunen–Loeve Transform) color spaces. The study in [89] analyzed only the Q value of the YIQ color scheme to determine the ROI section. Then, a composite space Y.I/Q of the YIQ color space was presented to extract bleeding features by computing the mean, median, skewness, and minima of the pixel values. Based on the YIQ color histogram, another article proposed an automatic bleeding detection scheme for CE images [90]. The YCbCr color space was presented to collect information from CE images in order to identify images with lesions [91]. Yuan et al., investigated several color histograms, including RGB, HSV, YCbCr, and CIE L*a*b*, and proposed the YCbCr color space to extract bleeding features for discrimination of bleeding images from normal CE images [92]. The studies in [93,94,95,96] suggested using the CIE L*a*b* color space for detection or localization of the bleeding region in CE images. Mathew et al. proposed a bleeding zone detection system based on the contourlet transform in the CIE XYZ color space [97]. Another feature extraction color space was the Karhunen-Loeve (K-L) Transform, which was utilized for fuzzy region segmentation in CE images [98]. The study by X. Liu et al. [99] proposed a computer-aided bleeding and ulcer detection approach based on the covariance of second-order statistical features in the K-L color space. A K-means color group was suggested as a color feature extractor for superpixel segmentation to find bleeding regions in CE videos [100].

**Combined multiple color spaces**: To detect bleeding, a group of color features was computed using multiple color spaces in CE images. The method described in [26] employed two distinct enhancing operations for identifying bleeding in CE images: the first was for the RGB color space, and the second was for the grayscale color space. The study in [101] determined the ROI of bleeding CE images using the YIQ color space and extracted features from the ROI using the CMYK color space. Based on the RGB and HSV color spaces, CE images were defined using statistical characteristics to extract bleeding features [33,102]. A 9-D feature was extracted at the superpixel level from the RGB and HSV color spaces during the segmentation stage of the study [103]. Some studies utilized a combination of the RGB and HSI color spaces to extract features for the bleeding detection approach in CE images [104,105,106,107]. Five color spaces (RGB, HSV, CIE L*a*b*, YCbCr, and CMYK) were used to extract features in [108]. In [7], the authors proposed the R channel with respect to the G and B channels and the ratio of G and B channels as features in the RGB color space, and the HSV color space was chosen for the saturation feature. The color features were extracted using the color components X = {R, G, B, L, a, b, H, S, V, F1, F2, F3} in the RGB, CIE L*a*b*, and HSV color spaces from each superpixel of CE images [109]. In [110], the color components H, S, a, b from the HSV and CIE L*a*b* color spaces and the Ros (Rosenfeld–Troy) metric were used. Ten features, including Normalized Excessive Red (NER), Hue, sum RGB, chroma, etc., were used to analyze CE video frames in [111]. For the segmentation of bleeding regions from bleeding CE images, delta E color differences were used to extract features by applying nine color shades (red, orange, brown, maroon, purple, pink, mahogany, brown, and bittersweet) for characterizing different types of bleeding [45]. The recommended Probability Density Function (PDF) fitting-based feature extraction technique was used in the YIQ, HSV, and CIE L*a*b* color spaces [112]. In [113], 40 features were extracted from five different channels, including R in the RGB color space, V in the HSV color space, Cr in the YCbCr color space, and a and L in the CIE L*a*b* color space. An article [114] investigated 21 color components from RGB, YUV, YIQ, HSB, CIE XYZ, and CIE L*a*b* color spaces for feature extraction, such as U/Y, V/Y, I/Y, and Q/Y. A. K. Kundu et al. [115] demonstrated a combination of the HSV and YIQ color spaces using normal PDF to detect GI diseases in CE videos. According to [30], the HSV color space was used for threshold analysis of the classification model, and the CIE L*a*b* color space was used in the trainable model for edge detection in images.

**Texture:** Texture feature is used to partition images into ROI and classify those regions as bleeding and non-bleeding. It provides information about an image in the spatial patterns of colors or intensities that repeat. In [75,116], a conventional texture representation model, named uniform Local Binary Pattern (LBP), was used to differentiate bleeding and normal regions. The study in [35] extracted the texture features (LBP) from suspicious areas in images and their surroundings for classifying bleeding. Zhao et al. [38] extracted an LBP based on the contourlet transform as texture features to segment abnormalities in WCE images. As a color texture feature, Li et al. [12] integrated chrominance moments and uniform LBP to discriminate bleeding regions from normal regions. Charfi et al. [88] also extracted texture features (LBP) for segmentation from WCE images in order to prevent false detections. For recognizing bleeding regions, a 6D color texture feature vector {x = (R, G, B, H, S, I)} was developed in [106]. Pogorelov et al. [4] presented bleeding detection system-computed texture features to extract additional information from the captured image frames. By using a histogram of the index image, a distinguishable color texture feature was developed in [53] for automatic bleeding image detection.

The Gray-Level Co-occurrence Matrix (GLCM) is a statistical approach for assessing texture that considers the spatial interaction of pixels. The GLCM functions describe the texture of CE images by computing how frequently pairs of pixels with given values and in a specified spatial relationship appear in an image, generating a GLCM, and afterward extracting statistical measures from this matrix. In [117], the authors proposed an efficient normalized GLCM for extracting the bleeding features from CE images. In [118], the authors proposed a texture feature descriptor-based algorithm that operated on the normalized GLCM of the magnitude spectrum of the images for a real-time computerized GI hemorrhage detection system. The study in [72] compared two types of texture features, GLCM and Homogeneous Texture Descriptor (HTD), with various numbers of color histogram bins. Rathnamala et al. [45] extracted texture attributes from the Gaussian mixture model superpixels in WCE images.

**Shape:** Shape feature extraction from images involves the process of identifying and describing the geometric characteristics of objects or regions within the image. This includes detecting object boundaries; computing features like area, perimeter, circularity, and eccentricity; and representing the shape using descriptors like chain codes, Hu moments, Histogram of Oriented Gradients (HOG), or Fourier descriptors. Among them, HOG is a popular shape feature extraction method commonly used for object detection and recognition tasks. The studies in [119,120,121] specifically utilized the HOG descriptor for shape feature extraction.

**Extraction Domain:** According to the taxonomy, the extraction domain is the process of extracting bleeding features from CE images. All of the reviewed studies were categorized into three parts depending on the extraction domain: global feature (when the features are extracted from the whole frames or images); local feature (when the features are extracted at the pixel-level or from a portion of an image -specific block size, ROI, POI); and combined local and global features (when the features are extracted at both pixel and image levels).

**Global feature:** The entire image information is used in the global feature extraction technique. Using statistical features (such as mean, mode, variance, moment, entropy, energy, skewness, kurtosis, etc.), several articles [1,30,61,71,78,79,94,96,103,109,115,116,122] extracted bleeding features from whole CE images. In [50], statistical color features of bleeding images were extracted from the RGB plane’s first-order histogram. In another study [79], statistical features were measured from GLCM after applying an Undecimated Double Density Dual Tree Discrete Wavelet Transform on CE images. Cui et al. [74] applied six color statistical features to identify bleeding features from the full image feature. Zhou et al. [73] utilized color information to extract the bleeding features from the image feature. In [9], color, shape, and surf were used for feature extraction from whole images.

**Local feature:** A pixel-level feature extraction approach was proposed in several studies in order to accurately identify bleeding images [29,36,51,56,58,120,121,123]. Instead of computing different features from each pixel, a few researchers proposed block-based local feature extraction techniques to reduce time and computational cost [37,47]. The study in [39] investigated various overlapping block sizes (3 × 3, 5 × 5, 7 × 7, and 9 × 9) and proposed a 7 × 7 block size to extract features from CE images. Maghsoudi et al. [80] divided the original image of 512 × 512 pixels into 256 sub-images with a resolution of 32 × 32 pixels for feature extraction. In [70], the 576 × 576 pixels input was sliced into nine non-overlapping blocks, each with 64 × 64 pixels. Another research group divided each CE image into blocks of 64 × 64 pixels and analyzed the 64 × 64 = 4096 pixels in each block to recognize hemorrhage [69]. CE images are surrounded by a large black background, which provides unwanted features. As a result, this reduces the performance of the model. To address this, a few studies introduced a Region Of Interest (ROI) for proper feature extraction. In [92,100,119], the authors selected an ROI from a maximum square inside the circular CE image without loss of main information. The ROI was 180 × 180 pixels in size, chosen from a total of 256 × 256 pixels. An elliptical ROI was selected inside the image to extract local features in [33]. According to [101], an ROI of the bleeding CE image was determined using the YIQ color space. After that, CMYK values were computed within the ROI pixels, which were applied to discriminate bleeding and non-bleeding pixels. An ROI was selected based on the Q-value of the YIQ color space and a composite space Y.I/Q was used to capture the bleeding information from the ROI section of the CE images [89]. The Pixel Of Interest (POI) technique can also extract local features that depend on the intensity values of pixels. In [112,115], the authors utilized POI instead of whole CE images to extract features for the classification of bleeding images.

**Combined local and global features:** Studies used local and global features to develop robust and accurate computer-aided bleeding detection systems. The studies in [65,106,107] proposed bleeding detection application software that was tested at the pixel and image levels. Region-level block-based and image-level global feature extraction techniques were applied in [39] to identify bleeding images. There were two stages presented in [4]; the first used only local color features to categorize bleeding images, while the second included global texture and color features to classify bleeding pixels. In [37], a block-based local feature extraction technique was presented, and then global features were extracted using a color histogram to classify bleeding and non-bleeding images. Ghosh et al. [57] presented an approach that used the maximum pixel value of each proposed spatial block and the global features to classify bleeding images. In another study [59], the authors used pixels to remove the edge zone and grouped pixels adaptively based on the red ratio in the RGB color space for superpixel segmentation. Another study proposed a global feature descriptor based on magnitude spectrum entropy and a local textural descriptor based on the contrast, sum entropy, sum variance, difference variance, and difference average, operating on the normalized GLCM [118]. Few researchers have proposed various machine learning and deep learning algorithms to extract both global and local features from CE images. The study in [22] proposed a bleeding detection method using a genetic algorithm for feature selection from CE images. Using an unsupervised K-means clustering algorithm, some studies extracted features for automatic bleeding detection in CE images [39,64]. Three pre-trained deep convolutional neural networks (CNNs), named ResNet50, VGG19, and InceptionV3 models, were used to extract features from CE images suggested by [23].

## 5. Algorithm

Initially, researchers proposed various threshold values to detect bleeding [51,52]. Various machine learning (ML) and deep learning (DL) algorithms are currently being used for accurate bleeding detection.

**Machine Learning (ML):** Several ML algorithms have been applied in computer-aided bleeding detection systems to effectively detect bleeding in CE images or videos, such as SVM, KNN, K-Means Clustering, Naïve Bayes, Random Tree, Random Forest, ANN, PNN, MLP, etc.

**SVM** is one of the most popular supervised ML algorithms that is used to detect bleeding and non-bleeding images or zones from CE images or videos. The majority of studies used SVM based on the extracted features of input images including color space and texture [119,122]. In 2008, Liu et al. [48] developed an automated obscure bleeding detection technique for the GI tract that could classify bleeding and non-bleeding CE images using the SVM algorithm. An automated bleeding detection approach was presented in [47] that provided an accuracy of 97.67%. Another research group suggested an automatic bleeding image detection technique utilizing an SVM classifier to detect bleeding and non-bleeding frames from CE videos. The approach reported 94.50% accuracy, 93.00% sensitivity, and 94.88% specificity [53]. The study in [36] utilized the SVM classifier to train with 200 bleeding and 200 non-bleeding CE images and achieved 97.96%, 97.75%, and 97.99% accuracy, sensitivity, and specificity, respectively. Studies in [49,124] suggested a system to automatically detect bleeding in CE images using the SVM classifier. A recent study suggested a Quadratic Support Vector Machine (QSVM) classifier for an automated bleeding detection approach, which was proposed in [1]. A fuzzy logic technique was applied to extract the features of the images. The model achieved 98.2%, 98%, and 98% accuracy, sensitivity, and specificity, respectively. Joshi et al. [125] presented an SVM classification model based on an improved Bag of Visual Words to detect bleeding in CE images.

Different kernel functions of the SVM algorithm, including linear, polynomial (cubic [126], quadratic [81]), and Radial Basis Function (RBF) [120] were used in bleeding detection in CE image research. The SVM classifier with the linear kernel was utilized in a real-time computerized gastrointestinal hemorrhage detection method for CE videos. The results obtained 99.19%, 99.41%, and 98.95% accuracy, sensitivity, and specificity, respectively [118]. Liu et al. [60] presented an automatic detection gastric hemorrhage system using the SVM classifier with RBF as the kernel function, which achieved 95.8% accuracy, 87.5% sensitivity, 98.1% specificity, 12.5% miss detection rate, and 1.9% false detection rate, respectively. Another study [21] also used the RBF kernel of the SVM classifier to discriminate between bleeding and non-bleeding images, achieving 98.0%, 97.0%, and 98.0% accuracy, specificity, and sensitivity, respectively. In addition [105], the SVM classifier model was used to detect bleeding using the chi-square kernel and histogram intersection. The combination of spatial pyramids with a robust hue histogram improved the accuracy by about 8%.

**KNN** is the second most popular supervised ML algorithm to detect bleeding in CE images or videos. The algorithm processes all existing pixels of CE images and classifies new pixels based on similarities. In [90], the KNN classifier was used to train with CE videos, achieving 97.50% accuracy, 94.33% sensitivity, and 98.21% specificity. Kundu et al. [62] employed a KNN model for detecting bleeding in CE images, which achieved an accuracy of 98.12%, a sensitivity of 94.98%, and a specificity of 98.55%. The article in [103] presented a bleeding detection approach for CE images that was compared to various ML algorithms, such as SVM, AdaBoost (Adaptive Boosting), and KNN, and the KNN algorithm achieved the best results with 99.22% accuracy. In another study, a KNN classifier was employed [71] to distinguish the characteristics of bleeding and non-bleeding images. The classifier was trained with 200 color CE images and achieved 99.0% accuracy.

**ANN or NN** is an ML algorithm for computer system designs that are inspired by biological neural networks. The approach described in [50] employed an ANN with 3 input neurons, 22 hidden neurons, and 2 output neurons, with a minimum squared error loss function. The ANN classifier was also applied in a preprocessing step to analyze the pixels in CE images in [25]. ANN is also known as NN. A NN cell classifier was applied in [76,96] to categorize bleeding and non-bleeding patches in CE images. Another study proposed back-propagation NN to detect bleeding regions, achieving 97% sensitivity and 90% specificity. A Probabilistic Neural Network (PNN) is a radial basis function and Bayesian theory-based feedforward neural network. One such study [107] applied PNN to detect bleeding zones in CE images, achieving 93.1% sensitivity and 85.6% specificity. Multilayer Perceptron (MLP) is a type of fully connected feedforward ANN that is widely used in statistical pattern recognition. Several articles [32,75,80,82,83] employed MLP neural networks to classify bleeding images and regions in CE images. The article in [114] proposed the Vector Supported Convex Hull classification algorithm, which was compared to SVM and configured with two alternative feature selection approaches. The model achieved a 98% sensitivity and specificity ratio for bleeding detection. Another study [102] suggested a computer-aided color feature-based bleeding detection technique using a modified ant colony optimization algorithm. The model achieved 98.82%, 99.66%, and 98.01% accuracy, sensitivity, and specificity, respectively.

**Other ML:** The Naïve Bayes classifier is another ML classification algorithm based on the Bayes Theorem. The studies in [54,100] used a Naïve Bayes classifier to detect bleeding in CE images. In [38,94], another ML algorithm, K-means clustering, was applied to extract important features for summarizing CE video clips. Random Tree and Random Forest are tree-based ML algorithms for making decisions. In the study in [78], a Random Tree classifier was trained with 100 bleeding and 100 non-bleeding images for a computer-aided bleeding detection system. The classifier achieved an accuracy of 99%, a sensitivity of 98%, and a specificity of 99%. In [79], both Random Tree and Random Forest classifier models outperformed bleeding detection compared to MLP and Naïve Bayes models. Both models provided 99.5%, 99%, and 100% accuracy, sensitivity, and specificity, respectively. The Random Forest model was also used in [77], achieving 95.7% sensitivity and 92.3% specificity. In [65,123], an Expectation Maximization (EM) clustering algorithm was used to detect potential bleeding regions as the ROI for subsequent classification of bleeding images from normal ones.

**Combined multiple ML:** In [110], a block-based segmentation technique using local features was presented and several ML algorithms, like linear discriminant analysis, SVM, Random Forest, and ADABoost, were applied for discriminating between bleeding and non-bleeding images. Using the SVM and K-means algorithms, a GI bleeding detection approach was presented in [64] to detect bleeding images and regions of bleeding images, reporting less computation time with 98.04% accuracy and 84.88% precision. According to [72], an automatic detection system was designed to identify suspected blood indicators in CE images. The authors compared various ML classifiers, like SVM and NN. The ISVM classifier was trained with 136 normal with 214 abnormal images and achieved a maximum of 98.13% accuracy using the Total Margin-Based Adaptive Fuzzy (TAF-SVM) algorithm. In addition, an automatic bleeding detection approach for CE videos was suggested by Ghosh et al. using a cluster-based feature. The SVM classifier was applied to the clustering information to detect bleeding zones in CE images, obtaining a precision of 97.05%, False Positive Rate (FPR) of 1.1%, and False Negative Rate (FNR) of 22.38% [39].

**Deep Learning (DL):** The most widely used deep learning approach for image classification and segmentation is the Convolutional Neural Network (CNN). Several CNN models, such as AlexNet [95], LeNet [127], Fully Convolutional Neural Network (FCN) [27,128], Visual Geometry Group Network (VGGNet) [129], Residual Network (Resnet-50) [130,131,132], Res2Net101 [133], Inception-Resnet-V2 [134,135], AttResU-Net [136], MobileNet [42], DenseNet [24], Region-based Convolutional Neural Networks (R-CNN) [137], Convolutional Recurrent Neural Network (CRNN) [34], U-Net [44,138], SegNet [46], and custom CNNs [41,139,140,141,142,143,144,145,146,147,148,149,150,151,152,153], have been utilized in a number of studies for the classification or segmentation or combined classification and segmentation of bleeding in CE images. The study in [27] presented an FCN model for an automatic blood region segmentation system. Another study [128] proposed a Look-Behind FCN algorithm for abnormality detection (polyps, ulcers, and blood) in CE images, achieving an accuracy of 97.84%. In [127], a LeNet model was trained and adopted pre-trained AlexNet, VGG-Net, and GoogLeNet models to identify intestinal hemorrhage. In [46], the authors applied a pre-trained AlexNet model for identification and a SegNet model for segmentation of intestinal bleeding. Another CNN algorithm, named U-Net, was proposed in [28,44] to segment bleeding areas in CE images and videos. Xing et al. [24] proposed a saliency-aware hybrid network algorithm based on two densely connected convolutional networks (DenseNets) for an automatic bleeding detection system. The authors of [85] developed a CNN model for detecting bleeding zones, which was trained using SegNet layers with bleeding, non-bleeding, and background classes. A blood content detection approach using ResNet architecture with 50 layers was suggested in [130], which achieved an accuracy of 99.89%, a sensitivity of 96.63%, and a specificity of 99.96%. Hwang et al. used a CNN model based on VGGNet to identify lesions with 96.83% accuracy [129]. Another CNN model for classifying bleeding in CE images was provided in another study [42]. The model was created utilizing MobileNet and a custom-built CNN. To identify small bowel angioectasia, the authors of [154] used a 16-layer Single Shot MultiBox Detector (SSD) deep CNN method.

**Combined ML & DL algorithms:** In recent years, several researchers have employed CNN-based models, such as AlexNet [126], VGG [86], ResNet [121,131], InceptionV3 [23], DenseNet [155], and XcepNet23 [81], to extract relevant features from medical images, particularly in tasks like identifying bleeding from normal images. In [86], deep CNNs (VGG16 and VGG19) were applied to extract features from CE images. A KNN algorithm was proposed to classify bleeding images, achieving 99.42% and 99.51% accuracy and precision rate, respectively. An automatic bleeding region segmentation technique was presented in [156] using individual MLP and CNN models. In another study [23], the authors proposed pre-trained deep CNNs (VGG19, InceptionV3, and ResNet50) models to extract bleeding features, and ML algorithms (SVM, KNN, Linear Regression) were utilized to distinguish bleeding and non-bleeding images. In [155], the authors applied DenseNet for feature extraction and the features were trained with an MLP algorithm to classify GI track abdominal infections. In addition, the study in [157] applied a CNN model to extract bleeding features and the SVM classifier was used to detect bleeding.

## 6. Discussion

**Figure 3:**
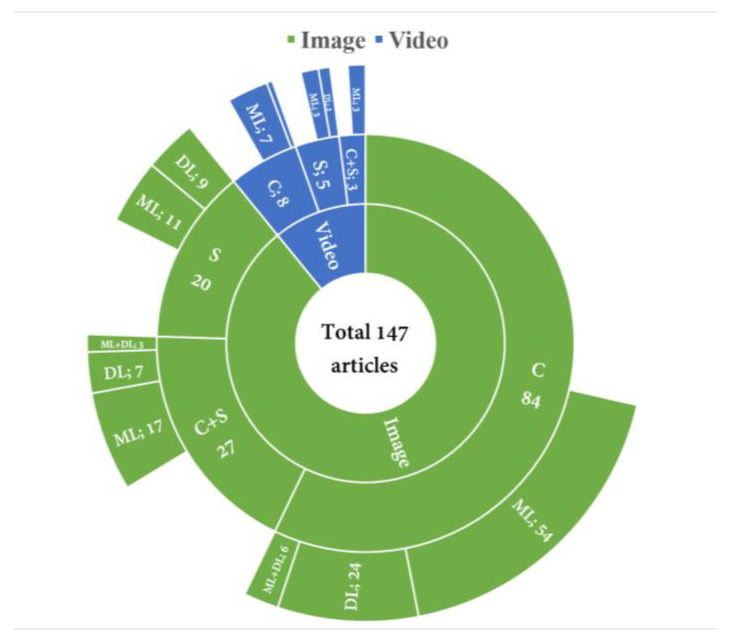
Overview of all studies in this review. C = classification, S = segmentation, C + S = combined both classification and segmentation, ML = machine learning, DL = deep learning, ML + DL = both machine learning and deep learning.

A CE device typically records video in the GI tract for around 8 h. Few studies utilized video to detect bleeding abnormalities. Figure 3 shows an overview of the used domains and algorithms in the papers that were reviewed in this study. To detect bleeding in CE images, researchers used three tasks, which included classification (C), segmentation (S), and combined classification and segmentation (C + S). Articles using the proposed classification task showed an average accuracy of 96.13% ± 3.04, sensitivity of 95.13% ± 4.25, and specificity of 95.63% ± 4.03. For the segmentation algorithms, the average accuracy was around 94.95% ± 4.11, sensitivity was 92.44% ± 9.16, and specificity was 95.86% ± 2.16. Articles that used the proposed combined task achieved an average accuracy of 97.12% ± 1.95, sensitivity of 94.12% ± 9.10, and specificity of 96.63% ± 4.15. Based on the above literature analysis, the combined task performed better. One significant benefit of the current methods is their ability to identify bleeding in CE images/frames and pinpoint the specific bleeding region. However, a limitation of these methods is their inability to measure the extent or depth of the bleeding area.

**Figure 4:**
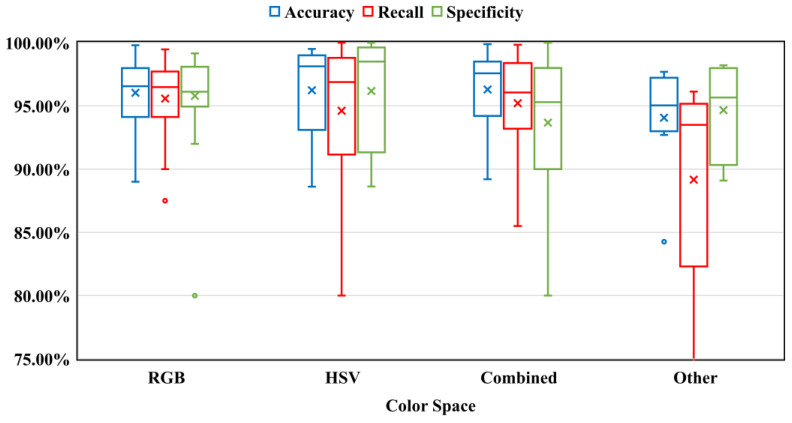
Performance of different color spaces. (‘X’ and circle represent mean and outlier, respectively).

Feature extraction is an essential part of bleeding detection in CE images. The feature values are extracted from the color channels of CE images. The performance of a bleeding detection algorithm directly depends on the feature values. To identify bleeding, many color spaces were presented. RGB is the popular color space to extract features because it is the default color space. Apart from the RGB color space, several studies proposed individual color channels (R, G, B, etc.), color channel pixel ratios (R/G, G/R, etc.), or various color spaces (HSV, YIQ, YCbCr, CIE L*a*b*, CIE XYZ, K-L, etc.) to extract appropriate bleeding features. A few researchers applied two or more color spaces together to extract features. According to the taxonomy, all of the suggested color spaces were categorized into four groups: RGB, HSV, Combined (multiple color spaces), and Other (YIQ, YCbCr, CIE L*a*b*, CIE XYZ, etc.). It is important to acknowledge that the performance results presented in this article are directly extracted from the original papers. The box plots in all the figures were used to compare the performance between groups of methods, rather than between individual algorithms. Statistical measures such as the mean, median, 25th percentile, and 75th percentile were utilized for each group. The performance results of different color spaces in detecting bleeding using a box plot are shown in Figure 4. According to the figure, the choice of color space did not provide any performance benefits. All of the color spaces provided similar results except the ‘Other’ color space. When comparing all color spaces, the RGB color space had a slightly higher recall value. It should be noted that the recall performance criterion is the most important in the detection of bleeding. On top of that, the RGB color space achieved lower variance for accuracy, recall, and specificity. The current methods make a significant contribution by investigating all potential color spaces to detect bleeding in capsule endoscopy.

**Figure 5:**
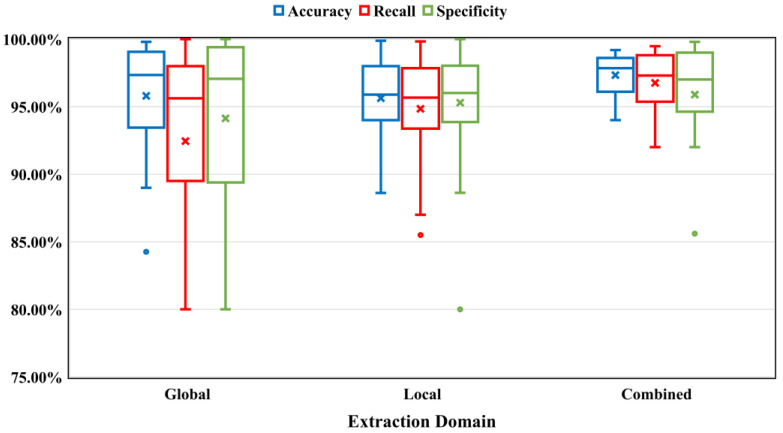
Performance of different feature extraction domains. (‘X’ and circle represent mean and outlier, respectively).

A typical approach for extracting bleeding features from whole CE images is the global feature extraction domain. Because this approach analyzes the entire image at once, the complexity and processing time are increased. The average results obtained using different extraction domains using a box plot are shown in Figure 5. To address the problem, various studies proposed a pixel-level feature extraction domain, in which the technique analyzed each pixel of the CE image. The technique improved the results but did not reduce the complexity and computation time. Several authors selected a portion of the CE image (specific block size, ROI, POI) in the preprocessing step for feature extraction, named the local feature extraction domain. The technique improved the results and reduced the complexity and processing time. Recently, a few researchers applied both the global and local feature extraction domains in a computer-aided bleeding detection system, which significantly enhanced the detection accuracy compared to the individual domains. Texture and statistical values (mean, mode, variance, moment, entropy, energy, skewness, kurtosis, etc.) were calculated using the feature values. Finally, a classification or segmentation algorithm was used to extract the values in order to detect bleeding in the CE images. According to Figure 5, the combined feature extraction domain outperformed the other domains in terms of accuracy, sensitivity, and specificity because it was tested at both the pixel and image levels. Also, a CNN model was used to train the model at the pixel level, followed by the application of a classification model to detect bleeding. The current feature extraction methods have certain limitations, such as introducing bias (which is influenced by the chosen algorithm), increased complexity, overfitting, and reduced generalizability.

**Figure 6:**
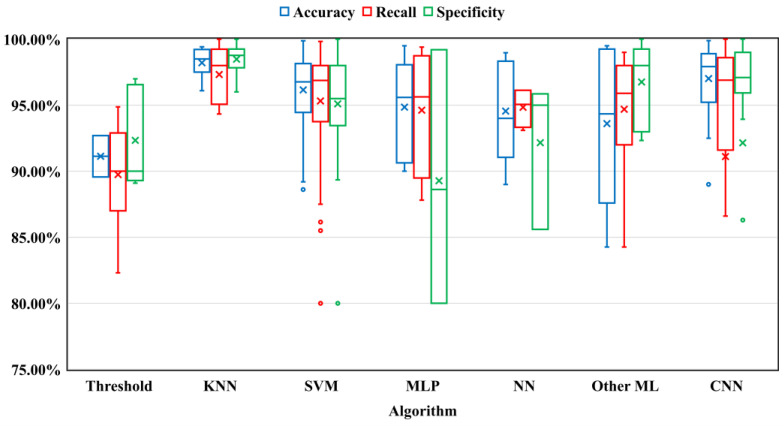
Performance of different state-of-the-art algorithms. (‘X’ and circle represent mean and outlier, respectively).

The majority of the literature reviewed proposed various ML algorithms that were trained with the texture and statistical features to identify bleeding in the CE images. Before introducing advanced ML algorithms, researchers set threshold values for the extraction features to detect bleeding in the CE images. The review of the literature found that the greatest number of articles used the KNN, SVM, MLP, and NN algorithms. In addition to these algorithms, a few other ML algorithms were used, such as Principal Component Analysis (PCA), Random Tree, Random Forest, Fuzzy C-Means, Expectation Maximization clustering, and Vector Supported Convex Hull, which were called “Other ML” in this study. The performance of the ML technique is based on the extracted features of color channels. The color channel intensity values overlapped between bleeding and non-bleeding pixels. As a result, utilizing ML approaches to distinguish bleeding from non-bleeding in CE images is problematic. In the last few years, researchers have proposed a DL technique, particularly using CNNs (AlexNet, LeNet, FCN, VGGNet, ResNet-50, MobileNet, U-Net, DenseNet, and SegNet) to identify bleeding in CE images. DL is an end-to-end classification and segmentation approach that extracts features automatically at the pixel level. Unlike ML, the DL approach does not require a separate feature extraction stage and it extracts features automatically to provide more efficient outcomes. The performance results for different state-of-the-art ML and DL algorithms using a box plot are shown in Figure 6. From the figure, it is observed that both the KNN and CNN algorithms outperformed the other algorithms. While the existing methods have made significant contributions to the development of classification and segmentation algorithms for detecting bleeding with satisfactory performance, they have often been tested on a limited number of test samples, such as images. Furthermore, deep learning algorithms have not yet incorporated attention mechanisms to further enhance their performance. We have added Appendix A at the end of our paper, which includes all of the information gathered from the reviewed articles in Table A1. We compared the performance of the research methods of the reviewed articles using established metrics such as accuracy, recall, specificity, Dice score, F1 score, Intersection over Union (IoU), etc.

The most commonly used color spaces in the available state-of-the-art bleeding detection algorithms for CE images are RGB, HSV, YIQ, YCbCr, CIE L*a*b*, CIE XYZ, K-L, etc. Among them, from the above review, we can see that the RGB color space had a slightly higher recall value and achieved lower variance for accuracy, recall, and specificity. A computer-aided system also improved the execution speed because no color conversion operations are required when using the RGB color space. So, for practical use, the RGB color space is the best option as there is no need to convert the data into other color domains. For the feature extraction method, the combined global and local feature extraction domain showed greater detection accuracy compared to the individual domains, which makes it more suitable for practical use. For bleeding detection in CE images, most of the literature proposed ML algorithms, which included SVM, KNN, PCA, MLP, NN, Random Tree, Random Forest, etc. Also, the greatest number of articles on DL used CNNs (FCN, SegNet, U-Net, DenseNet, ResNet50, VGGNet, and MobileNet). From the above review of the literature, the KNN and CNN algorithms outperformed the other algorithms. For ML algorithms, the color channel intensity values overlapped between bleeding and non-bleeding pixels. Meanwhile, the DL approach does not require a separate feature extraction stage and it extracts features automatically, which is more effective for practical use.

## 7. Limitations

To be suitable for practical use and deliver robust performance on unknown test data, the current methods require improvement. These improvements should include minimizing computational requirements and visually representing the outcomes to ensure that clinicians can trust the results. In the above review, the chosen color space did not provide any performance benefits for the classification or segmentation tasks. The RGB color space provided higher execution speed with lower performance variance, and it should be used in the future. DL algorithms provide a promising path for practical computer-aided bleeding detection systems in CE. However, DL algorithms depend on the number of medical image datasets and the insufficient quantity of data is a limitation.

## 8. Future Direction

Deep learning-based software like Enlitic’s Curie|ENDEX™ is being used to help radiologists manage and analyze medical imaging data in an efficient way [158]. In addition, Medtronic PillCam™ COLON CAD, Olympus CAD EYE™, and EndoBRAIN^®^ (Fujifilm, Tokyo, Japan) are currently being used by physicians to automatically detect GI abnormalities in CE images [159]. Although there are just few examples of computer-aided bleeding detection, it is promising as the technology is continuing to develop. More real-life effective algorithms are being introduced by researchers. This review paper will help guide them towards algorithms that could be used by practicing physicians. Recently, a new technique called a Generative Adversarial Network (GAN) has been introduced to generate synthetic images [160,161,162,163]. So, in the future, the dataset limitation may be overcome by collecting more data using synthetic CE images. Additionally, DL algorithms may be a better alternative for detecting bleeding in CE images. Several articles introduced ML and DL algorithms concurrently, such as DL for feature extraction and ML for bleeding image categorization. The integrated models considerably improved the performance of computer-aided bleeding detection systems. From the literature review, the majority of articles used the same patient or subject data for the training and testing datasets as they randomly selected images for training and testing. This introduces bias in an experimental setup. Same-patient CE images should not be mixed in training and testing datasets for classifying bleeding.

## 9. Conclusions

A systematic review of the available state-of-the-art computer-aided bleeding detection algorithms for capsule endoscopy (CE) was conducted, and the most accurate and suitable algorithms for practical use were identified. This review suggests a taxonomy for computer-aided bleeding detection systems. Researchers used various color spaces and feature extraction techniques to boost the bleeding detection algorithm performance. The analysis revealed that the choice of color space offered no additional benefits. For simplicity, the RGB color space is preferred. For the feature extraction, combining both global and local feature extraction domains in a computer-aided bleeding detection system significantly enhanced the detection accuracy compared to individual domains. The k-nearest neighbor (KNN) and convolutional neural network (CNN) outperformed the other algorithms for computer-aided bleeding detection systems. However, the KNN algorithm faces a few limitations, like overfitting and hand-crafted feature extraction. Recently, computer-aided bleeding detection systems have focused on deep learning algorithms. The performance of deep learning bleeding detection algorithms is improving day by day. In the future, deep learning algorithms will be a promising path for computer-aided bleeding detection systems in capsule endoscopy.

## Figures and Tables

**Table 1:** Eligibility Criteria.

Inclusion Criteria	Exclusion Criteria
Articles published in peer-reviewed venues.	Articles that do not involve bleeding, lesion, or hemorrhage.
Articles published from 1 January 2001 to 24 July 2023.	Articles not written in English.
Articles must address a set of keywords: *(Bleeding OR Hemorrhage OR blood) AND (Detection OR Segmentation OR Recognition OR Classification) AND (Capsule Endoscopy).*	Exclude articles on non-humans.
Articles that describe an automatic computer-aided bleeding detection system for capsule endoscopy.	Exclude posters and book chapters.

## Data Availability

Not applicable.

## Appendix A

**Table A1:** Summary of the reviewed state-of-the-art algorithms.

Refs.	Domain	Methodology/Technique			Feature Extraction		CS	DS	Results
		Task	ML/DL	Algorithm	Color Space	Extraction Domain			
2006 [65]	Image	C	ML	Expectation Maximization	RGB	Combined	-	201 images	R: 92%, S: 98%
2008 [35]	Video	C	ML	SVC	HSV	Local	10-fold	84 videos	A: ~97%
2008 [48]	Image	C	ML	SVM	RGB	Local	-	640 images	A: ~99%
2008 [66]	Video	C	ML	SVM	HSV	Global	-	5 videos	R: 80%
2008 [104]	Image	S	ML	Threshold	Combined	Global	-	2000 images	R: 92.86%, S: 89.49%
2008 [98]	Image	C + S	ML	Threshold	Other	Combined	-	-	-
2008 [75]	Image	C	ML	MLP	HSV	Local		3600 images	A: 90.84%
2009 [83]	Image	C	ML	MLP	HSV	Local	4-fold	100 images	R: 87.81% ± 1.36, S: 88.62% ± 0.44
2009 [82]	Image	C	ML	MLP	HSV	Local	-	200 images	Detection rate: 90%.
2009 [70]	Image	C	ML	SVM, NN	HSV	Local	10-fold	300 images	A: 99.41% (SVM), A: 98.97% (NN)
2009 [52]	Image	S	ML	Threshold	RGB	Global	-	4800 images	R: 94.87%, S: 96.12%
2009 [106]	Image	C	ML	NN	Combined	Combined	-	14,630 images	R: 93.03%, S: 95.86%
2010 [107]	Image	C	ML	PNN	Combined	Combined	-	14,630 images	R: 93.1%, S: 85.6%
2010 [74]	Image	C	ML	SVC	HSV	Global	-	6416 images	A: ~97%
2010 [38]	Video	S	ML	K-Means Clustering	RGB	Local	-	10 videos	-
2010 [76]	Image	C	ML	NN	HSV	Local	-	200 images	A: 93.1%
2011 [25]	Image	C	ML	ANN	RGB	Local	-	2000 images	A: 94%, R: 94%, S: 95%
2011 [105]	Image	C	ML	SVM	Combined	Combined	5-fold	560 images	A: 97.9%, R: 97.8%, S: 98.0%
2011 [7]	Image	C	ML	Threshold	Combined	Local	-	42 images	R: 87%, S: 90%
2012 [69]	Image	C	ML	Threshold	HSV	Local	-	72 images	In 3 images, the algorithm did not detect bleeding
2012 [49]	Image	C	ML	SVM	RGB	Local	-	52 images	-
2012 [72]	Image	C	ML	SVM	HSV	Local	5-fold	350 images	A: 98.13%
2012 [51]	Image	C	ML	Threshold	RGB	Local	-	14,630 images	R: 90%, S: 97%
2012 [99]	Image	C	ML	Threshold	Other	Global	-	100 images	R: 82.3%, S: 89.10%
2012 [114]	Image	C + S	ML	Vector Supported Convex Hull	Combined	Local	-	50 videos	R/S: >98%
2013 [111]	Image	C + S	ML	SVM	Combined	Local	-	10 videos	FPR: 4.03%
2013 [55]	Image	C	ML	SVM	RGB	Local	-	-	R rises to 0.8997
2013 [50]	Image	C	ML	ANN	RGB	Global	-	90 images	A: 89%
2013 [93]	Image	S	ML	Threshold	Other	Global	-	700 images	A: 92.7%, R: 92.9%
2014 [53]	Image	C	ML	SVM	RGB	Local	-	2250 images	A: 94.50%, R: 93.00%, S: 94.88%
2014 [58]	Image	C	ML	SVM	RGB	Local	-	200 images	A: 95.80%, R: 96.50%, S: 95.63%
2014 [59]	Image	C + S	ML	SVM	RGB	Combined	10-fold	5000 images	A: 94%, R: 97%, S: 92%
2014 [56]	Image	C	ML	KNN	RGB	Local	1-fold	200 images	A: 98.5%, R: 98.0%, S: 99.0%
2014 [73]	Image	C	ML	SVM	HSV	Global	5-fold	1413 images	A: 95.33%, R: 96.88%, S: 89.35%
2014 [71]	Image	C	ML	KNN	HSV	Global	1-fold	200 images	A: 99.0%, R: 100.0%, S: 98.0%
2014 [101]	Image	C	ML	SVM	Combined	Local	1-fold	1000 images	A: 93.40%, R: 95.50%, S: 92.87%
2014 [32]	Video	C + S	ML	MLP	RGB	Global	-	428 images	A: 93.7%, R: 94.5%, S: 80.0%
2015 [78]	Image	C	ML	Random Tree	HSV	Global	10-fold	200 images	A: 99%, R: 98%, S: 99%
2015 [118]	Image	C	ML	SVM	RGB	Combined	-	1200 images	A: 99.19%, R: 99.41%, S: 98.95%
2015 [63]	Image	C	ML	SVM	RGB	Local	-	800 images	A: 95.89%, R: 98.77%, S: 93.45%
2015 [61]	Image	S	ML	Threshold	RGB	Global	-	690 images	A: 89.56%
2015 [57]	Image	C	ML	KNN	RGB	Combined	1-fold	1000 images	A: 96.10%, R: 96.48%, S: 96.01%
2015 [89]	Image	C	ML	SVM	Other	Local	1-fold	15 videos	A: 93.90%, R: 93.50%, S: 94%
2015 [90]	Image	C	ML	KNN	Other	Local	10-fold	2300 images	A: 97.50%, R: 94.33%, S: 98.21%
2015 [97]	Image	C	ML	KNN	Other	Local	10-fold	332 images	A: 96.38%, R: 95.17%, S: 97.32%
2015 [92]	Image	C	ML	SVM	Other	Local	10-fold	2400 images	A: 95.75%, AUC: 0.9771
2015 [109]	Image	C	ML	SVM	Combined	Global	10-fold	252 images	AUC: 94%, R: 96%, S: 91%
2015 [26]	Image	S	ML	SVM	Combined	Local	-	3596 images	A: 94.10%, R: 91.69%, S: 94.59%
2016 [91]	Image	S	ML	PCA	Other	Local	10-fold	1330 images	A: 94.34% ± 0.0235, AUC: 0.9532 ± 0.0172
2016 [31]	Video	S	ML	Threshold	RGB	Local	-	15 videos	-
2016 [62]	Video	C + S	ML	KNN	RGB	Local	10-fold	2300 images	A: 98.12%, R: 94.98%, S: 98.55%
2016 [64]	Image	C + S	ML	SVM	RGB	Local	-	10,000 images	R: 96.88%, P: 99.23%, F1: 98.04%
2016 [40]	Video	S	ML	SVM	RGB	Local	8-fold	8 videos	A: 97%, R: 95.83%, S: 98.08%
2016 [87]	Image	C	ML	SVM	HSV	Local	5-fold	1650 images	A: 88.61%
2016 [79]	Image	C	ML	Random Tree, Random Forest	HSV	Global	10-fold	200 images	A: 99.5%, R: 99%, S: 100%
2016 [108]	Image	C	ML	SVM	Combined	Local	10-fold	400 images	A: 89.2%, R: 93.5%, S: 80%
2016 [102]	Image	C + S	ML	SVM	Combined	Local	5-fold	300 images	A: 98.82%, R: 99.66%, S: 98.01%
2016 [157]	Image	C	DL+ ML	(CNN); SVM	-	-	-	10,000 images	R: 99.20%, P: 99.90%, F1: 99.55%
2016 [125]	Image	C	ML	SVM	-	Global	4-fold	912 images	A: 95.06%
2017 [80]	Image	C + S	ML	MLP	HSV	Local	-	223 images	A: 97.47%
2017 [29]	Video	C	ML	SVC	HSV	Local	10-fold	30 videos	A: 92%, R: 94%, S: 91%
2017 [47]	Image	C	ML	SVM	RGB	Local	-	1200 images	A: 97.67%, R: 97.57%, S: 95.46%
2017 [36]	Video	C + S	ML	SVM	RGB	Local	10-fold	400 images	A: 97.96%, R: 97.75%, S: 97.99%, T: 0.280 sec
2017 [21]	Image	C	ML	SVM	RGB	Local	-	400 images	A: 98%, R: 97%, S: 98%
2017 [88]	Image	C + S	ML	SVM	HSV	Local	3-fold	970 images	A: 94.4%
2017 [43]	Image	S	ML	SVM	HSV	Local	-	50 BL images	-
2017 [68]	Image	S	ML	Threshold	HSV	Local	-	401 images	R: 88.3%,
2017 [33]	Video	C	ML	SVM	Combined	Local	5-fold	8872 images	A: 95%, R: 94% S: 95.3%
2017 [127]	Image	C	DL	LeNet, AlexNet, VGG-Net, GoogLeNet	Other	-	-	12,090 images	F1: 98.87%
2017 [27]	Image	C + S	DL	FCN	-	-		300 images	IoU: 0.7750
2017 [164]	Image	C	DL	CNN	RGB	-	-	1500 images	R: 91%, P: 94.79%, F1: 92.85%
2018 [128]	Image	C	DL	FCN	RGB	-	10-fold	10,000 images	A: 97.84%, AUC: 99.72%
2018 [85]	Image	S	DL	SegNet	HSV	-	-	335 images	A: 94.42%, IoU: 90.69%
2018 [165]	Image	C	DL	CNN	Other	-	-	-	AUC: 0.90
2018 [41]	Video	S	DL	CNN	-	-	-	6360 images	R: 100%, S: 96%
2018 [28]	Video	S	DL	U-Net	-	-	-	3295 images	ACC: 95.88%, R: 99.56%, S: 93.93%
2018 [116]	Image	S	ML	SVM	Combined	Global	-	50 BL images	Dice: 0.81, ME: 0.092
2018 [9]	Image	C + S	ML	SVM	HSV	Global	10-fold	412 images	A: 98.49%, T: 17.393 sec
2018 [37]	Video	C	ML	KNN	RGB	Combined	10-fold	32 videos	A: 97.85%, R: 99.47%, S: 99.15%, P: 95.75%
2018 [39]	Video	C	ML	SVM	RGB	Combined	10-fold	32 videos	P: 97.05%, FPR: 1.1%, FNR: 22.38
2018 [54]	Image	C + S	ML	Naive Baïes	RGB	Local	-	-	M:0.3478, SD:0.3306 for R channel.
2018 [67]	Image	C	ML	Fuzzy C-Means	HSV	Local	10-fold	1275 images	A: 90.92%
2018 [103]	Image	C + S	ML	KNN	Combined	Global	-	1000 images	A: 99.22%, R: 98.51%, S: 99.53%
2019 [117]	Image	C	ML	SVM	RGB	Local	5-fold	3500 images	A: 92.45%, R: 90.76%, S: 94.65%
2019 [4]	Image	C	ML	SVM	RGB	Combined	10-fold	1200 images	A: 97.7%, R: 97.6%, S: 95.5%, F1: 97.8%, MCC: 89.8%
2019 [60]	Image	C + S	ML	SVM	RGB	Local	5-fold	240 images	A: 95.8%, R: 87.5%, S: 98.1%
2019 [77]	Image	C	ML	Random Forest	HSV	Local	5-fold	75 images	R: 95.68%, S: 92.33%
2019 [11]	Image	C	ML	KNN	HSV	Local	10-fold	2393 images	A: 98.8%, R: 99%, S: 99%, P: 95%, F1: 97%
2019 [113]	Image	C + S	ML	SVM	Combined	Local	10-fold	3895 images	A: 98.2%, FPR: 02.5%, P: 97.5%, R: 98.8%, F: 98.2%, MCC: 96.3%, F1: 98.2%
2019 [110]	Image	C + S	ML	SVM	Combined	Local	10-fold	90 images	A: 95%, R: 85%, S: 97%
2019 [112]	Image	C	ML	SVM	Combined	Local	10-fold	30 videos	A: 96.77%, R: 97.55%, S: 96.59%
2019 [154]	Image	C	DL	CNN	-	-	-	2237 images	AUC: 99.8%, R: 98.8%, S: 98.4%, PPV: 75.4%, NPV: 99.9%
2019 [22]	Image	C	ML	MLP	Combined	Local	-	3895 images	A: 97.58%, R: 96.76%, P: 98.29%
2019 [24]	Image	C	DL	DenseNet	-	Local	-	1200 images	R: 97.3%, P: 94.7%, F1: 95.9%
2019 [130]	Image	C	DL	ResNet50	-	-	-	27,847 images	AUC: 0.9998, A: 99.89%, R: 96.63%, S: 99.96%,
2019 [155]	Image	C + S	DL + ML	(DenseNet); MLP	Combined	-	10-fold	12,000 images	A: 99.5%, R: 99.40%, S: 99.20%,
2019 [44]	Image	S	DL	U-Net	-	-	-	335 images	A: 98.5%, IoU: 86.3%
2019 [84]	Image	S	DL	CNN	HSV	-	5-fold	778 images	A: 98.9%, R: 94.8%, S: 99.1%, AUC: 99.7%
2019 [86]	Image	C + S	DL + ML	(CNN); KNN	HSV	Global	10-fold	4500 images	A: 99.42%, P: 99.51%, S: 100%
2019 [156]	Image	C + S	DL + ML	(CNN); MLP	Combined	Global	-	778 images	AUC-ROC: >0.97
2020 [129]	Image	C	DL	VGGNet	-	-	10-fold	7556 images	A: 96.83%, R: 97.61%, S: 96.04%
2020 [166]	Image	C + S	DL	CNN	Combined	-	-	94 images	A: 97.8%, R: 98.6%, S: 86.3%
2020 [167]	Image	S	DL	CNN	Other	-	-	3895 images	A: 95%
2020 [115]	Image	C	ML	SVM	Combined	Global	10-fold	2588 images	A: 92.23%, R: 86.15%, P: 89.11, F1: 97.60
2020 [100]	Image	C + S	ML	Naive Baïes	Other	Local	-	55,000 images	-
2020 [124]	Image	C	ML	SVM	-	Local	5-fold	912 images	A: 98.18%, R: 98%, P: 98%, F1: 98%
2021 [46]	Image	C + S	DL	SegNet	HSV	-	-	2350 images	A: 94.42%, IoU: 90.69%
2021 [30]	Video	C	ML	SVM	Combined	Global	-	2 videos	-
2021 [23]	Image	C	DL + ML	(VGG19, InceptionV3, ResNet50); SVM	RGB	Global	10-fold	56,448 images	A: 97.71%
2021 [1]	Image	C	ML	SVM	HSV	Global	10-fold	2393 images	A: 98.2%, R: 98%, S: 98%, P: 93%, F1: 95.4%
2021 [45]	Image	C + S	ML	SVM	Combined	Local	10-fold	3294 images	A: 99.88%, R: 99.83%, S: 100%
2021 [42]	Image	C	DL	MobileNet	-	-	10-fold	1650 images	A: 99.3%, P: 100%, R: 99.4%, F1: 99.7%
2021 [138]	Image	S	DL	U-Net	RGB	-	-	3295 images	A: 95.90%, Dice: 91%
2021 [140]	Image	C	DL	CNN	RGB	-	-	23,720 images	R: 86.6%, S: 95.9%
2021 [141]	Image	C + S	DL	CNN	RGB	-	-	20,000 images	A: 98.9%, F1: 93.5%
2021 [137]	Image	C + S	DL	RCNN	RGB	-	-	1302 images	R: 66.67%, P: 85.71%, F1: 75.00%
2021 [135]	Image	C	DL	Inception-Resnet-V2	RGB	-	-	400,000 images	A: 98.07%, AUC: 92.2%,
2021 [142]	Image	C	DL	CNN	RGB	-	-	11,588 images	R: 91.8%, S: 95.9%
2021 [95]	Image	C	DL	AlexNet	Other	-	-	420 images	A: 94.5%, R: 95.24%, S: 96.72%
2021 [143]	Image	C	DL	CNN	RGB	-	-	5825 images	R: 99.8%, S: 93.2%
2021 [126]	Image	C	DL + ML	(AlexNet); SVM	RGB	Global	-	24,000 images	A: 99.8%
2021 [144]	Image	C + S	DL	CNN	RGB	-	5-fold	77 images	A: 98%, IoU: 81%, Dice: 56%
2021 [119]	Image	C	ML	SVM	Combined	Local	10-fold	3895 images	A: 95.4%, P: 95.6%, R: 95.2%
2021 [145]	Image	C	DL	CNN	RGB	-	-	53,555 images	A: 99%, R: 88%, S: 99%
2021 [123]	Image	S	ML	Expectation Maximization	Combined	Local	10-fold	3895 images	P: 96.5%, R: 95.9%, S: 93.2%
2022 [120]	Image	C	ML	SVM	RGB	Local	-	3895 images	P: 98.11%, R: 98.55%
2022 [146]	Image	C	DL	CNN	RGB	-	-	6130 images	A:95.6%, R:90.8%, S: 97.1%
2022 [96]	Image	C	ML	NN	Other	Global	-	1,722,499 images	A: 97.69%, P: 96.47%, R: 96.13%
2022 [132]	Image	C + S	DL	ResNet-50	RGB	-	4-fold	4900 images	A: 95.1%
2022 [133]	Image	S	DL	Res2Net101	RGB	Combined	-	1136 images	IoU: 86.86%
2022 [168]	Image	C	DL + ML	(CNN); PCA, SVM	RGB	Global	-	912 images	A: 95.62%, P: 95.7%, R: 95.62%, F1: 95.62%
2022 [147]	Image	C	DL	CNN	RGB	-	-	1200 images	A: 98.5%, R: 98.5%, F1: 98.5%, AUC: 99.49%
2022 [148]	Image	C	DL	CNN	RGB	-	-	49,180 images	R: 93.4%, S: 97.8%
2022 [149]	Image	C	DL	CNN	RGB	-	-	21,320 images	A: 97.1%, R: 95.9%, S: 97.1%
2022 [150]	Image	C	DL	CNN	RGB	-	-	9005 images	R: 99.8%, S: 100.0%
2022 [151]	Image	C	DL	CNN	-	-	-	5000 images	A: 99.3%, P: 100%, S: 99.4%
2022 [152]	Image	S	DL	CNN	RGB	-	-	48 images	Dice: 69.91%
2022 [134]	Image	C	DL	Inception-ResNet-V2	RGB	-	9-fold	3895 images	A: 98.5%, R: 98.5%, S: 99.0%
2022 [153]	Image	C	DL	CNN	RGB	-	-	22,095 images	A: 98.5%, R: 98.6%, S:98.9%
2022 [34]	Video	C	DL	CRNN	RGB	-	-	240 videos	A: 89%, R: 97%
2023 [136]	Image	S	DL	AttResU-Net	RGB	-	-	3295 images	A: 99.16%, Dice: 94.91%, IoU: 90.32%
2023 [81]	Image	C	DL + ML	(ResNet18, XcepNet23); Q_SVM	HSV	Combined	5-fold	4000 images	A: 98.60%, R: 98.60%, S: 99.80%
2023 [131]	Image	S	DL	Resnet-50	RGB	-	-	12,403 images	A: 99%, IoU: 69%
2023 [122]	Image	C + S	ML	SVM; KNN	-	Global	-	-	A: 95.75%, AUC: 97.71%
2023 [94]	Image	S	ML	K-Means Clustering	Other	Global	-	48 images	A: 84.26%, R:69.84%, Dice: 67.71%
2023 [139]	Image	C	DL	CNN	RGB	-	-	18,625 images	A: 92.5%, R: 96.8%, S: 96.5%
2023 [121]	Image	C	DL + ML	(ResNet-50); SVM	Combined	Local	-	5689 images	A: 97.82%, R: 97.8%, F1: 97.8%

DS = datasets, C = classification, S = segmentation, C + S = combined both classification and segmentation, ML = machine learning, DL = deep learning, DL + ML = both deep learning and machine learning, BL = bleeding, A = accuracy, R = recall = sensitivity, S = specificity, Dice = Dice score, F1 = F1 score, IoU = intersection over union, AUC = area under the curve, ROC = receiver operator characteristic, PPV = positive predictive value, NPV = negative predictive value, FPR = false positive rate, FNR = false negative rate, MCC = matthews correlation coefficient, ME = misclassification error, and T = execution time.

## References

[1] Al Mamun A., Em P.P., Ghosh T., Hossain M.M., Hasan M.G., Sadeque M.G. (2021). Bleeding recognition technique in wireless capsule endoscopy images using fuzzy logic and principal component analysis. Int. J. Electr. Comput. Eng..

[2] Monteiro S., De Castro F.D., Carvalho P.B., Moreira M.J., Rosa B., Cotter J. (2016). PillCam^®^ SB3 capsule: Does the increased frame rate eliminate the risk of missing lesions?. World J. Gastroenterol..

[3] Fan S., Xu L., Fan Y., Wei K., Li L. (2018). Computer-aided detection of small intestinal ulcer and erosion in wireless capsule endoscopy images. Phys. Med. Biol..

[4] Pogorelov K., Suman S., Azmadi Hussin F., Saeed Malik A., Ostroukhova O., Riegler M., Halvorsen P., Hooi Ho S., Goh K.-L. (2019). Bleeding detection in wireless capsule endoscopy videos—Color versus texture features. J. Appl. Clin. Med. Phys..

[5] Karargyris A., Bourbakis N. (2011). Detection of small bowel polyps and ulcers in wireless capsule endoscopy videos. IEEE Trans. Biomed. Eng..

[6] Zuckerman G.R., Prakash C., Askin M.P., Lewis B.S. (2000). AGA technical review on the evaluation and management of occult and obscure gastrointestinal bleeding. Gastroenterology.

[7] Lee Y., Yoon G., Architecture A.O. (2011). Bleeding detection algorithm for capsule endoscopy. World Acad. Sci. Eng. Technology.

[8] Park S.C., Chun H.J., Kim E.S., Keum B., Seo Y.S., Kim Y.S., Jeen Y.T., Lee H.S., Um S.H., Kim C.D. (2012). Sensitivity of the suspected blood indicator: An experimental study. World J. Gastroenterol..

[9] Liaqat A., Khan M.A., Shah J.H., Sharif M., Yasmin M., Fernandes S.L. (2018). Automated ulcer and bleeding classification from wce images using multiple features fusion and selection. J. Mech. Med. Biol..

[10] Al Mamun A., Hossain M.S., Em P.P., Tahabilder A., Sultana R., Islam M.A. (2021). Small intestine bleeding detection using color threshold and morphological operation in WCE images. Int. J. Electr. Comput. Eng..

[11] Al Mamun A., Hossain M.S., Hossain M.M., Hasan M.G. (2019). Discretion Way for Bleeding Detection in Wireless Capsule Endoscopy Images. Proceedings of the 1st International Conference on Advances in Science, Engineering and Robotics Technology 2019.

[12] Neumann H., Fry L.C., Nägel A., Neurath M.F. (2014). Wireless capsule endoscopy of the small intestine: A review with future directions. Curr. Opin. Gastroenterol..

[13] Karargyris A., Bourbakis N. (2010). Wireless Capsule Endoscopy and Endoscopic Imaging: A Survey on Various Methodologies Presented. IEEE Eng. Med. Biol. Mag..

[14] Brito H.P., Ribeiro I.B., de Moura D.T.H., Bernardo W.M., Chaves D.M., Kuga R., Maahs E.D., Ishida R.K., de Moura E.T.H., de Moura E.G.H. (2018). Video capsule endoscopy vs double-balloon enteroscopy in the diagnosis of small bowel bleeding: A systematic review and meta-analysis. World J. Gastrointest. Endosc..

[15] Koulaouzidis A., Rondonotti E., Karargyris A. (2013). Small-bowel capsule endoscopy: A ten-point contemporary review. World J. Gastroenterol..

[16] Iakovidis D.K., Koulaouzidis A. (2015). Software for enhanced video capsule endoscopy: Challenges for essential progress. Nat. Rev. Gastroenterol. Hepatol..

[17] Hwang Y., Park J., Lim Y.J., Chun H.J. (2018). Application of artificial intelligence in capsule endoscopy: Where are we now?. Clin. Endosc..

[18] Chen Y., Lee J. (2012). A review of machine-vision-based analysis of wireless capsule endoscopy video. Diagn. Ther. Endosc..

[19] Shah N., Chen C., Montano N., Cave D., Siegel R., Gentile N.T., Limkakeng A.T., Kumar A.B., Ma Y., Meltzer A.C. (2020). Video capsule endoscopy for upper gastrointestinal hemorrhage in the emergency department: A systematic review and meta-analysis. Am. J. Emerg. Med..

[20] Soffer S., Klang E., Shimon O., Nachmias N., Eliakim R., Ben-Horin S., Kopylov U., Barash Y. (2020). Deep learning for wireless capsule endoscopy: A systematic review and meta-analysis. Gastrointest. Endosc..

[21] Suman S., Hussin F.A.B., Walter N., Malik A.S., Ho S.H., Goh K.L. (2017). Detection and classification of bleeding using statistical color features for wireless capsule endoscopy images. Proceedings of the 2016 International Conference on Signal and Information Processing, IConSIP 2016.

[22] Amiri Z., Hassanpour H., Beghdadi A. (2019). Feature Selection for Bleeding Detection in Capsule Endoscopy Images using Genetic Algorithm. Proceedings of the 5th Iranian Conference on Signal Processing and Intelligent Systems, ICSPIS 2019.

[23] Caroppo A., Leone A., Siciliano P. (2021). Deep transfer learning approaches for bleeding detection in endoscopy images. Comput. Med. Imaging Graph..

[24] Xing X., Yuan Y., Jia X., Max Q.H.M. A saliency-aware hybrid dense network for bleeding detection in wireless capsule endoscopy images. Proceedings of the 2019 IEEE 16th International Symposium on Biomedical Imaging (ISBI 2019).

[25] Fu Y., Mandal M., Guo G. Bleeding region detection in WCE images based on color features and neural network. Proceedings of the 2011 IEEE 54th International Midwest Symposium on Circuits and Systems (MWSCAS).

[26] Xiong Y., Zhu Y., Pang Z., Ma Y., Chen D., Wang X. (2015). Bleeding detection in wireless capsule endoscopy based on MST clustering and SVM. Proceedings of the IEEE Workshop on Signal Processing Systems, SiPS: Design and Implementation.

[27] Jia X., Meng M.Q.-H. (2017). A study on automated segmentation of blood regions in Wireless Capsule Endoscopy images using fully convolutional networks. Proceedings of the International Symposium on Biomedical Imaging.

[28] Coelho P., Pereira A., Leite A., Salgado M., Cunha A. (2018). A Deep Learning Approach for Red Lesions Detection in Video Capsule Endoscopies. Lecture Notes in Computer Science.

[29] Usman M.A., Satrya G.B., Usman M.R., Shin S.Y. (2016). Detection of small colon bleeding in wireless capsule endoscopy videos. Comput. Med. Imaging Graph..

[30] Usman M.A., Usman M.R., Satrya G.B., Khan M.A., Politis C., Philip N., Shin S.Y. (2021). QI-BRiCE: Quality index for bleeding regions in capsule endoscopy videos. Comput. Mater. Contin..

[31] Novozámský A., Flusser J., Tachecí I., Sulík L., Bureš J., Krejcar O. (2016). Automatic blood detection in capsule endoscopy video. J. Biomed. Opt..

[32] Sainju S., Bui F.M., Wahid K.A. (2014). Automated bleeding detection in capsule endoscopy videos using statistical features and region growing. J. Med. Syst..

[33] Deeba F., Islam M., Bui F.M., Wahid K.A. (2018). Performance assessment of a bleeding detection algorithm for endoscopic video based on classifier fusion method and exhaustive feature selection. Biomed. Signal Process. Control.

[34] Ding Z., Shi H., Zhang H., Zhang H., Tian S., Zhang K., Cai S., Ming F., Xie X., Liu J. (2022). Artificial intelligence-based diagnosis of abnormalities in small-bowel capsule endoscopy. Endoscopy.

[35] Mackiewicz M., Fisher M., Jamieson C. Bleeding detection in wireless capsule endoscopy using adaptive colour histogram model and support vector classification. Proceedings of the Progress in Biomedical Optics and Imaging—Proceedings of SPIE.

[36] Ghosh T., Fattah S.A., Wahid K.A. (2018). Automatic Computer Aided Bleeding Detection Scheme for Wireless Capsule Endoscopy (WCE) Video Based on Higher and Lower Order Statistical Features in a Composite Color. J. Med. Biol. Eng..

[37] Ghosh T., Fattah S.A., Wahid K.A. (2018). CHOBS: Color Histogram of Block Statistics for Automatic Bleeding Detection in Wireless Capsule Endoscopy Video. IEEE J. Transl. Eng. Health Med..

[38] Zhao Q., Meng M.Q.-H., Li B. WCE video clips segmentation based on abnormality. Proceedings of the 2010 IEEE International Conference on Robotics and Biomimetics, ROBIO 2010.

[39] Ghosh T., Fattah S.A., Wahid K.A., Zhu W.-P., Ahmad M.O. (2018). Cluster based statistical feature extraction method for automatic bleeding detection in wireless capsule endoscopy video. Comput. Biol. Med..

[40] Shi W., Chen J., Chen H., Peng Q., Gan T. (2016). Bleeding fragment localization using time domain information for WCE videos. Proceedings of the 2015 8th International Conference on BioMedical Engineering and Informatics, BMEI 2015.

[41] Leenhardt R., Vasseur P., Li C., Saurin J.C., Rahmi G., Cholet F., Becq A., Marteau P., Histace A., Dray X. (2019). A neural network algorithm for detection of GI angiectasia during small-bowel capsule endoscopy. Gastrointest. Endosc..

[42] Rustam F., Siddique M.A., Siddiqui H.U.R., Ullah S., Mehmood A., Ashraf I., Choi G.S. (2021). Wireless Capsule Endoscopy Bleeding Images Classification Using CNN Based Model. IEEE Access.

[43] Tuba E., Tuba M., Jovanovic R. An algorithm for automated segmentation for bleeding detection in endoscopic images. Proceedings of the International Joint Conference on Neural Networks.

[44] Li S., Zhang J., Ruan C., Zhang Y. Multi-Stage Attention-Unet for Wireless Capsule Endoscopy Image Bleeding Area Segmentation. Proceedings of the 2019 IEEE International Conference on Bioinformatics and Biomedicine, BIBM 2019.

[45] Rathnamala S., Jenicka S. (2021). Automated bleeding detection in wireless capsule endoscopy images based on color feature extraction from Gaussian mixture model superpixels. Med. Biol. Eng. Comput..

[46] Ghosh T., Chakareski J. (2021). Deep Transfer Learning for Automated Intestinal Bleeding Detection in Capsule Endoscopy Imaging. J. Digit. Imaging.

[47] Suman S., Hussin F.A.B., Malik A.S., Pogorelov K., Riegler M., Ho S.H., Hilmi I., Goh K.L. Detection and classification of bleeding region in WCE images using color feature. Proceedings of the ACM International Conference Proceeding Series.

[48] Liu J., Yuan X. (2009). Obscure bleeding detection in endoscopy images using support vector machines. Optim. Eng..

[49] Li J., Ma J., Tillo T., Zhang B., Lim E.G. A training based Support Vector Machine technique for blood detection in wireless capsule endoscopy images. Proceedings of the 2012 IEEE-EMBS Conference on Biomedical Engineering and Sciences, IECBES 2012.

[50] Sainju S., Bui F.M., Wahid K. Bleeding detection in wireless capsule endoscopy based on color features from histogram probability. Proceedings of the Canadian Conference on Electrical and Computer Engineering.

[51] Pan G., Xu F., Chen J. (2012). Bleeding detection in wireless capsule endoscopy using color similarity coefficient. Appl. Mech. Mater..

[52] Yun S.J., Young H.K., Dong H.L., Sang H.L., Jeong J.S., Jong H.K. Automatic patient-adaptive bleeding detection in a capsule endoscopy. Proceedings of the Progress in Biomedical Optics and Imaging—Proceedings of SPIE.

[53] Ghosh T., Fattah S.A., Shahnaz C., Wahid K.A. An automatic bleeding detection scheme in wireless capsule endoscopy based on histogram of an RGB-indexed image. Proceedings of the 36th Annual International Conference of the IEEE Engineering in Medicine and Biology Society.

[54] Sivakumar P., Kumar B.M. (2019). A novel method to detect bleeding frame and region in wireless capsule endoscopy video. Cluster Comput..

[55] Ma J., Tillo T., Zhang B., Wang Z., Lim E.G. Novel training and comparison method for blood detection in wireless capsule endoscopy images. Proceedings of the International Symposium on Medical Information and Communication Technology.

[56] Ghosh T., Bashar S.K., Alam M.S., Wahid K., Fattah S.A. A statistical feature based novel method to detect bleeding in wireless capsule endoscopy images. Proceedings of the 2014 International Conference on Informatics, Electronics and Vision, ICIEV 2014.

[57] Ghosh T., Fattah S.A., Shahnaz C., Kundu A.K., Rizve M.N. (2016). Block based histogram feature extraction method for bleeding detection in wireless capsule endoscopy. Proceedings of the IEEE Region 10 Annual International Conference, Proceedings/TENCON.

[58] Ghosh T., Fattah S.A., Wahid K.A. Automatic bleeding detection in wireless capsule endoscopy based on RGB pixel intensity ratio. Proceedings of the 1st International Conference on Electrical Engineering and Information and Communication Technology.

[59] Fu Y., Zhang W., Mandal M., Meng M.Q.-H. (2014). Computer-aided bleeding detection in WCE video. IEEE J. Biomed. Health Inform..

[60] Liu Z., Hu C., Shen Z. Research on a new feature detection algorithm for wireless capsule endoscope bleeding images based on super-pixel segmentation. Proceedings of the IEEE International Conference on Robotics and Biomimetics, ROBIO 2019.

[61] Kumar S., Figueiredo I.N., Graca C., Falcao G. (2015). A GPU accelerated algorithm for blood detection inwireless capsule endoscopy images. Lecture Notes in Computational Vision and Biomechanics.

[62] Kundu A.K., Rizve M.N., Ghosh T., Fattah S.A. (2017). A segmented color plane histogram based feature extraction scheme for automatic bleeding detection in wireless capsule endoscopy. Proceedings of the 2016 IEEE Students’ Technology Symposium, TechSym 2016.

[63] Yuan Y., Meng M.Q.H. Automatic bleeding frame detection in the wireless capsule endoscopy images. Proceedings of the IEEE International Conference on Robotics and Automation.

[64] Jia X., Cai L., Liu J., Dai W., Meng M.Q.-H. GI bleeding detection in wireless capsule endoscopy images based on pattern recognition and a MapReduce framework. Proceedings of the 2016 IEEE International Conference on Real-Time Computing and Robotics, RCAR 2016.

[65] Hwang S., Oh J., Cox J., Tang S.J., Tibbals H.F. Blood detection in wireless capsule endoscopy using expectation maximization clustering. Proceedings of the Progress in Biomedical Optics and Imaging—Proceedings of SPIE.

[66] Giritharan B., Yuan X., Liu J., Buckles B., Oh J., Tang S.J. Bleeding detection from capsule endoscopy videos. Proceedings of the 30th Annual International Conference of the IEEE Engineering in Medicine and Biology Society.

[67] Bchir O., Ben Ismail M.M., AlZahrani N. (2019). Multiple bleeding detection in wireless capsule endoscopy. Signal Image Video Process..

[68] Lau P.Y., Correia P.L. Detection of bleeding patterns in WCE video using multiple features. Proceedings of the 29th Annual International Conference of the IEEE Engineering in Medicine and Biology Society.

[69] Kukushkin A., Dmitry M., Ivanova E., Evgeny F., Zhukov I.U., Sergey S., Anastasia T., Rami M., Andrey S. Recognition of hemorrhage in the images of wireless capsule endoscopy. Proceedings of the Mediterranean Electrotechnical Conference—MELECON.

[70] Poh C.K., Zhang Z., Liang Z.Y., Li L., Liu J. Feature selection and classification for wireless capsule endoscopic frames. Proceedings of the International Conference on Biomedical and Pharmaceutical Engineering.

[71] Ghosh T., Bashar S.K., Fattah S.A., Shahnaz C., Wahid K.A. An automatic bleeding detection scheme in wireless capsule endoscopy based on statistical features in hue space. Proceedings of the 2014 17th International Conference on Computer and Information Technology, ICCIT 2014.

[72] Timotius I.K., Miaou S.-G., Valdeavilla E.B., Liu Y.-H. (2012). Abnormality detection for capsule endoscope images based on support vector machines. Biomed. Eng. Appl. Basis Commun..

[73] Zhou S., Song X., Siddique M.A., Xu J., Zhou P. (2015). Bleeding detection in wireless capsule endoscopy images based on binary feature vector. Proceedings of the 5th International Conference on Intelligent Control and Information Processing, ICICIP 2014.

[74] Cui L., Hu C., Zou Y., Meng M.Q.-H. Bleeding detection in wireless capsule endoscopy images by support vector classifier. Proceedings of the 2010 IEEE International Conference on Information and Automation, ICIA 2010.

[75] Li B., Meng M.Q.-H. Computer aided detection of bleeding in capsule endoscopy images. Proceedings of the Canadian Conference on Electrical and Computer Engineering.

[76] Poh C.K., Htwe T.M., Li L., Shen W., Liu J., Lim J.H., Chan K.L., Tan P.C. Multi-level local feature classification for bleeding detection in Wireless Capsule Endoscopy images. Proceedings of the 2010 IEEE Conference on Cybernetics and Intelligent Systems, CIS 2010.

[77] Pons P., Noorda R., Nevárez A., Colomer A., Beltrán V.P., Naranjo V. (2019). Design and Development of an Automatic Blood Detection System for Capsule Endoscopy Images. Lecture Notes in Computer Science.

[78] Dilna C., Gopi V.P. (2016). A novel method for bleeding detection in Wireless Capsule Endoscopic images. Proceedings of the 2015 International Conference on Computing and Network Communications, CoCoNet 2015.

[79] Reeha K.R., Shailaja K., Gopi V.P. Undecimated Complex Wavelet Transform based bleeding detection for endoscopic images. Proceedings of the 2016 2nd International Conference on Cognitive Computing and Information Processing, CCIP 2016.

[80] Maghsoudi O.H., Alizadeh M., Mirmomen M. (2017). A computer aided method to detect bleeding, tumor, and disease regions in Wireless Capsule Endoscopy. Proceedings of the 2016 IEEE Signal Processing in Medicine and Biology Symposium.

[81] Naz J., Sharif M.I., Sharif M.I., Kadry S., Rauf H.T., Ragab A.E. (2023). A Comparative Analysis of Optimization Algorithms for Gastrointestinal Abnormalities Recognition and Classification Based on Ensemble XcepNet23 and ResNet18 Features. Biomedicines.

[82] Li B., Meng M.Q.-H. (2009). Computer-aided detection of bleeding regions for capsule endoscopy images. IEEE Trans. Biomed. Eng..

[83] Li B., Meng M.Q.-H. (2009). Computer-based detection of bleeding and ulcer in wireless capsule endoscopy images by chromaticity moments. Comput. Biol. Med..

[84] Hajabdollahi M., Esfandiarpoor R., Najarian K., Karimi N., Samavi S., Reza Soroushmehr S.M. Low Complexity CNN Structure for Automatic Bleeding Zone Detection in Wireless Capsule Endoscopy Imaging. Proceedings of the 41st Annual International Conference of the IEEE Engineering in Medicine and Biology Society.

[85] Ghosh T., Li L., Chakareski J. Effective Deep Learning for Semantic Segmentation Based Bleeding Zone Detection in Capsule Endoscopy Images. Proceedings of the International Conference on Image Processing.

[86] Sharif M., Attique Khan M., Rashid M., Yasmin M., Afza F., Tanik U.J. (2019). Deep CNN and geometric features-based gastrointestinal tract diseases detection and classification from wireless capsule endoscopy images. J. Exp. Theor. Artif. Intell..

[87] Yuan Y., Li B., Meng M.Q.-H. (2017). WCE abnormality detection based on saliency and adaptive locality-constrained linear coding. IEEE Trans. Autom. Sci. Eng..

[88] Charfi S., El Ansari M. Gastrointestinal tract bleeding detection from wireless capsule endoscopy videos. Proceedings of the ACM International Conference Proceeding Series.

[89] Ghosh T., Fattah S.A., Bashar S.K., Shahnaz C., Wahid K.A., Zhu W.-P., Ahmad M.O. An automatic bleeding detection technique in wireless capsule endoscopy from region of interest. Proceedings of the International Conference on Digital Signal Processing DSP.

[90] Kundu A.K., Rizve M.N., Ghosh T., Fattah S.A., Shahnaz C. (2016). A histogram based scheme in YIQ domain for automatic bleeding image detection from wireless capsule endoscopy. Proceedings of the 2015 IEEE International WIE Conference on Electrical and Computer Engineering, WIECON-ECE 2015.

[91] Liu D.-Y., Gan T., Rao N.-N., Xing Y.-W., Zheng J., Li S., Luo C.-S., Zhou Z.-J., Wan Y.-L. (2016). Identification of lesion images from gastrointestinal endoscope based on feature extraction of combinational methods with and without learning process. Med. Image Anal..

[92] Yuan Y., Li B., Meng M.Q.-H. (2016). Bleeding Frame and Region Detection in the Wireless Capsule Endoscopy Video. IEEE J. Biomed. Health Inform..

[93] Figueiredo I.N., Kumar S., Leal C., Figueiredo P.N. (2013). Computer-assisted bleeding detection in wireless capsule endoscopy images. Comput. Methods Biomech. Biomed. Eng. Imaging Vis..

[94] Seebutda A., Sakuncharoenchaiya S., Numpacharoen K., Wiwatwattana N., Charoen A., Charoenpong T. Bleeding Region Segmentation in Wireless Capsule Endoscopy Images by K-Mean Clustering Technique. Proceedings of the 2023 Third International Symposium on Instrumentation, Control, Artificial Intelligence and Robotics (ICA-SYMP).

[95] Sunitha S., Sujatha S.S. An Improved Bleeding Detection Method for Wireless Capsule Endoscopy (WCE) Images Based on AlexNet. Proceedings of the 2021 3rd International Conference on Signal Processing and Communication (ICPSC).

[96] Lu B. (2022). Image Aided Recognition of Wireless Capsule Endoscope Based on the Neural Network. J. Healthc. Eng..

[97] Mathew M., Gopi V.P. Transform based bleeding detection technique for endoscopic images. Proceedings of the 2nd International Conference on Electronics and Communication Systems, ICECS 2015.

[98] Karargyris A., Bourbakis N. A methodology for detecting blood-based abnormalities in wireless capsule endoscopy videos. Proceedings of the 8th IEEE International Conference on BioInformatics and BioEngineering.

[99] Liu X., Gu J., Xie Y., Xiong J., Qin W. A new approach to detecting ulcer and bleeding in wireless capsule endoscopy images. Proceedings of the IEEE-EMBS International Conference on Biomedical and Health Informatics: Global Grand Challenge of Health Informatics, BHI 2012.

[100] Priyadharshini B., Gomathi T. (2020). Navie bayes classifier for wireless capsule endoscopy video to detect bleeding frames. Int. J. Sci. Technol. Res..

[101] Ghosh T., Bashar S.K., Fattah S.A., Shahnaz C., Wahid K.A. (2015). A feature extraction scheme from region of interest of wireless capsule endoscopy images for automatic bleeding detection. Proceedings of the 2014 IEEE International Symposium on Signal Processing and Information Technology, ISSPIT 2014.

[102] Mohammed S.K., Deeba F., Bui F.M., Wahid K.A. Application of modified ant colony optimization for computer aided bleeding detection system. Proceedings of the International Joint Conference on Neural Networks.

[103] Xing X., Jia X., Meng M.-H.Q. Bleeding Detection in Wireless Capsule Endoscopy Image Video Using Superpixel-Color Histogram and a Subspace KNN Classifier. Proceedings of the 2018 40th Annual International Conference of the IEEE Engineering in Medicine and Biology Society (EMBC).

[104] Jung Y.S., Kim Y.H., Lee D.H., Kim J.H. Active blood detection in a high resolution capsule endoscopy using color spectrum transformation. Proceedings of the 1st International Conference on BioMedical Engineering and Informatics, BMEI 2008.

[105] Lv G., Yan G., Wang Z. Bleeding detection in wireless capsule endoscopy images based on color invariants and spatial pyramids using support vector machines. Proceedings of the 2011 Annual International Conference of the IEEE Engineering in Medicine and Biology Society.

[106] Pan G., Yan G., Song X., Qiu X. (2009). BP neural network classification for bleeding detection in wireless capsule endoscopy. J. Med. Eng. Technol..

[107] Pan G., Yan G., Qiu X., Cui J. (2010). Bleeding detection in Wireless Capsule Endoscopy based on Probabilistic Neural Network. J. Med. Syst..

[108] Mohammed S.K., Deeba F., Bui F.M., Wahid K.A. Feature selection using modified ant colony optimization for wireless capsule endoscopy. Proceedings of the 2016 IEEE 7th Annual Ubiquitous Computing, Electronics and Mobile Communication Conference, UEMCON 2016.

[109] Iakovidis D.K., Chatzis D., Chrysanthopoulos P., Koulaouzidis A. Blood detection in wireless capsule endoscope images based on salient superpixels. Proceedings of the 2015 37th Annual International Conference of the IEEE Engineering in Medicine and Biology Society (EMBC).

[110] Obukhova N., Motyko A., Timofeev B., Pozdeev A. Method of endoscopic images analysis for automatic bleeding detection and segmentation. Proceedings of the Conference of Open Innovation Association, FRUCT.

[111] Yi S., Jiao H., Xie J., Mui P., Leighton J.A., Pasha S., Rentz L., Abedi M. A clinically viable Capsule Endoscopy video analysis platform for automatic bleeding detection. Proceedings of the SPIE—The International Society for Optical Engineering.

[112] Kundu A.K., Fattah S.A. (2019). Probability density function based modeling of spatial feature variation in capsule endoscopy data for automatic bleeding detection. Comput. Biol. Med..

[113] Amiri Z., Hassanpour H., Beghdadi A. A Computer- Aided Method to Detect Bleeding Frames in Capsule Endoscopy Images. Proceedings of the European Workshop on Visual Information Processing.

[114] Szczypiński P., Klepaczko A., Pazurek M., Daniel P. (2014). Texture and color based image segmentation and pathology detection in capsule endoscopy videos. Comput. Methods Programs Biomed..

[115] Kundu A.K., Fattah S.A., Wahid K.A. (2020). Least Square Saliency Transformation of Capsule Endoscopy Images for PDF Model Based Multiple Gastrointestinal Disease Classification. IEEE Access.

[116] Tuba E., Tomic S., Beko M., Zivkovic D., Tuba M. Bleeding Detection in Wireless Capsule Endoscopy Images Using Texture and Color Features. Proceedings of the 2018 26th Telecommunications Forum (TELFOR).

[117] Ponnusamy R., Sathiamoorthy S. (2019). An efficient gastrointestinal hemorrhage detection and diagnosis model for wireless capsule endoscopy. Int. J. Recent Technol. Eng..

[118] Hassan A.R., Haque M.A. (2015). Computer-aided gastrointestinal hemorrhage detection in wireless capsule endoscopy videos. Comput. Methods Programs Biomed..

[119] Amiri Z., Hassanpour H., Beghdadi A. (2021). A Computer-Aided Method for Digestive System Abnormality Detection in WCE Images. J. Healthc. Eng..

[120] Goyal A., Kaur J., Dhatarwal J., Handa P., Goel N. Automatic detection of WCE bleeding frames using hybrid features and machine learning algorithms. Proceedings of the 2022 IEEE India Council International Subsections Conference (INDISCON).

[121] Amiri Z., Hassanpour H., Beghdadi A. (2023). Combining deep features and hand-crafted features for abnormality detection in WCE images. Multimed. Tools Appl..

[122] Vajravelu A., Selvan K.S.T., Jamil M.M.B.A., Jude A., Diez I.D.L.T. (2023). Machine learning techniques to detect bleeding frame and area in wireless capsule endoscopy video. J. Intell. Fuzzy Syst..

[123] Amiri Z., Hassanpour H., Beghdadi A. (2022). Feature extraction for abnormality detection in capsule endoscopy images. Biomed. Signal Process. Control.

[124] Patel A., Rani K., Kumar S., Figueiredo I.N., Figueiredo P.N. (2020). Automated bleeding detection in wireless capsule endoscopy images based on sparse coding. Multimed. Tools Appl..

[125] Joshi I., Kumar S., Figueiredo I.N. (2016). Bag of visual words approach for bleeding detection in wireless capsule endoscopy images. Lecture Notes in Computer Science.

[126] Nayyar Z., Attique Khan M., Alhussein M., Nazir M., Aurangzeb K., Nam Y., Kadry S., Irtaza Haider S. (2021). Gastric Tract Disease Recognition Using Optimized Deep Learning Features. Comput. Mater. Contin..

[127] Li P., Li Z., Gao F., Wan L., Yu J. Convolutional neural networks for intestinal hemorrhage detection in wireless capsule endoscopy images. Proceedings of the Proceedings—IEEE International Conference on Multimedia and Expo.

[128] Diamantis D.E., Iakovidis D.K., Koulaouzidis A. (2019). Look-behind fully convolutional neural network for computer-aided endoscopy. Biomed. Signal Process. Control.

[129] Hwang Y., Lee H.H., Park C., Tama B.A., Kim J.S., Cheung D.Y., Chung W.C., Cho Y.-S., Lee K.-M., Choi M.-G. (2020). Improved classification and localization approach to small bowel capsule endoscopy using convolutional neural network. Dig. Endosc..

[130] Aoki T., Yamada A., Kato Y., Saito H., Tsuboi A., Nakada A., Niikura R., Fujishiro M., Oka S., Ishihara S. (2020). Automatic detection of blood content in capsule endoscopy images based on a deep convolutional neural network. J. Gastroenterol. Hepatol..

[131] Chu Y., Huang F., Gao M., Zou D.-W., Zhong J., Wu W., Wang Q., Shen X.-N., Gong T.-T., Li Y.-Y. (2023). Convolutional neural network-based segmentation network applied to image recognition of angiodysplasias lesion under capsule endoscopy. World J. Gastroenterol..

[132] Muruganantham P., Balakrishnan S.M. (2022). Attention Aware Deep Learning Model for Wireless Capsule Endoscopy Lesion Classification and Localization. J. Med. Biol. Eng..

[133] Li S., Si P., Zhang Z., Zhu J., He X., Zhang N. (2022). DFCA-Net: Dual Feature Context Aggregation Network for Bleeding Areas Segmentation in Wireless Capsule Endoscopy Images. J. Med. Biol. Eng..

[134] Garbaz A., Lafraxo S., Charfi S., El Ansari M., Koutti L. Bleeding classification in Wireless Capsule Endoscopy Images based on Inception-ResNet-V2 and CNNs. Proceedings of the 2022 IEEE Conference on Computational Intelligence in Bioinformatics and Computational Biology (CIBCB).

[135] Kim S.H., Hwang Y., Oh D.J., Nam J.H., Kim K.B., Park J., Song H.J., Lim Y.J. (2021). Efficacy of a comprehensive binary classification model using a deep convolutional neural network for wireless capsule endoscopy. Sci. Rep..

[136] Lafraxo S., Souaidi M., El Ansari M., Koutti L. (2023). Semantic Segmentation of Digestive Abnormalities from WCE Images by Using AttResU-Net Architecture. Life.

[137] Vieira P.M., Freitas N.R., Lima V.B., Costa D., Rolanda C., Lima C.S. (2021). Multi-pathology detection and lesion localization in WCE videos by using the instance segmentation approach. Artif. Intell. Med..

[138] Kanakatte A., Ghose A. Precise Bleeding and Red lesions localization from Capsule Endoscopy using Compact U-Net. Proceedings of the 2021 43rd Annual International Conference of the IEEE Engineering in Medicine & Biology Society (EMBC).

[139] Mascarenhas Saraiva M., Afonso J., Ribeiro T., Ferreira J., Cardoso H., Andrade P., Gonçalves R., Cardoso P., Parente M., Jorge R. (2023). Artificial intelligence and capsule endoscopy: Automatic detection of enteric protruding lesions using a convolutional neural network. Rev. Española Enfermedades Dig..

[140] Afonso J., Saraiva M.J.M., Ferreira J.P.S., Cardoso H., Ribeiro T., Andrade P., Parente M., Jorge R.N., Saraiva M.M., Macedo G. (2021). Development of a Convolutional Neural Network for Detection of Erosions and Ulcers with Distinct Bleeding Potential in Capsule Endoscopy. Tech. Innov. Gastrointest. Endosc..

[141] Lu F., Li W., Lin S., Peng C., Wang Z., Qian B., Ranjan R., Jin H., Zomaya A.Y. (2021). Multi-Scale Features Fusion for the Detection of Tiny Bleeding in Wireless Capsule Endoscopy Images. ACM Trans. Internet Things.

[142] Ribeiro T. (2021). Artificial intelligence and capsule endoscopy: Automatic detection of vascular lesions using a convolutional neural network. Ann. Gastroenterol..

[143] Mascarenhas Saraiva M., Ferreira J.P.S., Cardoso H., Afonso J., Ribeiro T., Andrade P., Parente M.P.L., Jorge R.N., Macedo G. (2021). Artificial intelligence and colon capsule endoscopy: Automatic detection of blood in colon capsule endoscopy using a convolutional neural network. Endosc. Int. Open.

[144] Jain S., Seal A., Ojha A., Yazidi A., Bures J., Tacheci I., Krejcar O. (2021). A deep CNN model for anomaly detection and localization in wireless capsule endoscopy images. Comput. Biol. Med..

[145] Mascarenhas Saraiva M.J., Afonso J., Ribeiro T., Ferreira J., Cardoso H., Andrade A.P., Parente M., Natal R., Mascarenhas Saraiva M., Macedo G. (2021). Deep learning and capsule endoscopy: Automatic identification and differentiation of small bowel lesions with distinct haemorrhagic potential using a convolutional neural network. BMJ Open Gastroenterol..

[146] Afonso J., Saraiva M.M., Ferreira J.P.S., Cardoso H., Ribeiro T., Andrade P., Parente M., Jorge R.N., Macedo G. (2022). Automated detection of ulcers and erosions in capsule endoscopy images using a convolutional neural network. Med. Biol. Eng. Comput..

[147] Biradher S., Aparna P. Classification of Wireless Capsule Endoscopy Bleeding Images using Deep Neural Network. Proceedings of the 2022 IEEE Delhi Section Conference (DELCON).

[148] Hosoe N., Horie T., Tojo A., Sakurai H., Hayashi Y., Limpias Kamiya K.J.-L., Sujino T., Takabayashi K., Ogata H., Kanai T. (2022). Development of a Deep-Learning Algorithm for Small Bowel-Lesion Detection and a Study of the Improvement in the False-Positive Rate. J. Clin. Med..

[149] Afonso J., Mascarenhas M., Ribeiro T., Cardoso H., Andrade P., Ferreira J.P.S., Saraiva M.M., Macedo G. (2022). Deep Learning for Automatic Identification and Characterization of the Bleeding Potential of Enteric Protruding Lesions in Capsule Endoscopy. Gastro Hep Adv..

[150] Mascarenhas M., Ribeiro T., Afonso J., Ferreira J.P.S., Cardoso H., Andrade P., Parente M.P.L., Jorge R.N., Mascarenhas Saraiva M., Macedo G. (2022). Deep learning and colon capsule endoscopy: Automatic detection of blood and colonic mucosal lesions using a convolutional neural network. Endosc. Int. Open.

[151] Goel S., Kumar Shah A. CNN-based Classification over Wireless Capsule Endoscopy Bleeding Images. Proceedings of the 2022 Second International Conference on Advanced Technologies in Intelligent Control, Environment, Computing & Communication Engineering (ICATIECE).

[152] Duangchai R., Toonmana C., Numpacharoen K., Wiwatwattana N., Charoen A., Charoenpong T. Bleeding Region Segmentation in Wireless Capsule Endoscopy Images by a Deep Learning Model: Initial Learning Rate and Epoch Optimization. Proceedings of the 2022 International Conference on Decision Aid Sciences and Applications (DASA).

[153] Mascarenhas Saraiva M., Ribeiro T., Afonso J., Ferreira J.P.S., Cardoso H., Andrade P., Parente M.P.L., Jorge R.N., Macedo G. (2022). Artificial Intelligence and Capsule Endoscopy: Automatic Detection of Small Bowel Blood Content Using a Convolutional Neural Network. GE Port. J. Gastroenterol..

[154] Tsuboi A., Oka S., Aoyama K., Saito H., Aoki T., Yamada A., Matsuda T., Fujishiro M., Ishihara S., Nakahori M. (2020). Artificial intelligence using a convolutional neural network for automatic detection of small-bowel angioectasia in capsule endoscopy images. Dig. Endosc..

[155] Khan M.A., Sharif M., Akram T., Yasmin M., Nayak R.S. (2019). Stomach Deformities Recognition Using Rank-Based Deep Features Selection. J. Med. Syst..

[156] Hajabdollahi M., Esfandiarpoor R., Khadivi P., Soroushmehr S.M.R., Karimi N., Najarian K., Samavi S. (2019). Segmentation of bleeding regions in wireless capsule endoscopy for detection of informative frames. Biomed. Signal Process. Control.

[157] Jia X., Meng M.Q.-H. A deep convolutional neural network for bleeding detection in Wireless Capsule Endoscopy images. Proceedings of the 2016 38th Annual International Conference of the IEEE Engineering in Medicine and Biology Society (EMBC).

[158] Mori Y., Kudo S., Berzin T.M., Misawa M., Takeda K. (2017). Computer-aided diagnosis for colonoscopy. Endoscopy.

[159] Kamitani Y., Nonaka K., Isomoto H. (2022). Current Status and Future Perspectives of Artificial Intelligence in Colonoscopy. J. Clin. Med..

[160] Zhuang P., Schwing A.G., Koyejo O. FMRI data augmentation via synthesis. Proceedings of the 2019 IEEE 16th International Symposium on Biomedical Imaging (ISBI 2019).

[161] Zhao H., Li H., Maurer-Stroh S., Cheng L. (2018). Synthesizing retinal and neuronal images with generative adversarial nets. Med. Image Anal..

[162] Zhao H., Li H., Cheng L. (2017). Synthesizing Filamentary Structured Images with GANs. arXiv.

[163] Bellemo V., Burlina P., Yong L., Wong T.Y., Ting D.S.W. (2019). Generative Adversarial Networks (GANs) for Retinal Fundus Image Synthesis. Lecture Notes in Computer Science.

[164] Jia X., Meng M.Q.-H. Gastrointestinal Bleeding Detection in Wireless Capsule Endoscopy Images Using Handcrafted and CNN Features. Proceedings of the 2017 39th Annual International Conference of the IEEE Engineering in Medicine and Biology Society (EMBC).

[165] Vasilakakis M.D., Diamantis D., Spyrou E., Koulaouzidis A., Iakovidis D.K. (2020). Weakly Supervised Multilabel Classification for Semantic Interpretation of Endoscopy Video Frames. Evol. Syst..

[166] Hajabdollahi M., Esfandiarpoor R., Sabeti E., Karimi N., Soroushmehr S.M.R., Samavi S. (2020). Multiple Abnormality Detection for Automatic Medical Image Diagnosis Using Bifurcated Convolutional Neural Network. Biomed. Signal Process. Control.

[167] Pannu H.S., Ahuja S., Dang N., Soni S., Malhi A.K. (2020). Deep Learning Based Image Classification for Intestinal Hemorrhage. Multimed. Tools Appl..

[168] Rani K., Devi G., Kumar S., Figueiredo I.N., Figueiredo P.N. Classification of Wireless Capsule Endoscopy Images for Bleeding Using Deep Features Fusion. Proceedings of the 2022 International Conference on Electrical, Computer, Communications and Mechatronics Engineering (ICECCME).

